# Sagittal Synostosis and Its Association With Cognitive, Behavioral, and Psychological Functioning: A Meta-analysis

**DOI:** 10.1001/jamanetworkopen.2021.21937

**Published:** 2021-09-13

**Authors:** Amanda J. Osborn, Rachel M. Roberts, Diana S. Dorstyn, Ben G. Grave, David J. David

**Affiliations:** 1 School of Psychology, The University of Adelaide, Adelaide, South Australia, Australia; 2 Craniofacial Australia, North Adelaide, South Australia, Australia

## Abstract

**Importance:**

Findings on the cognitive, behavioral, and psychological functioning of individuals with sagittal synostosis (SS) are highly disparate, limiting their clinical utility.

**Objective:**

To identify and review research on individuals with SS and to determine whether, and to what extent, they experience cognitive, behavioral, and psychological difficulties compared with their healthy peers or normative data for each measure.

**Data Sources:**

PubMed, Scopus, Embase, and PsycINFO were searched through January 2021 with no date restrictions. Scopus citation searches and manual checks of the reference lists of included studies were conducted.

**Study Selection:**

Studies included participants of any age who had received a diagnosis of single-suture (isolated or nonsyndromic) SS or scaphocephaly and who had been assessed on cognitive, behavioral, and psychological outcomes.

**Data Extraction and Synthesis:**

Data were independently extracted by 2 reviewers. Case-control outcomes (individuals with SS vs healthy peers or normative data) were compared using random-effects models with 3 effect sizes calculated: weighted Hedges *g* (*g_w_*), odds ratios (ORs), and mean prevalence rates. This study follows the Meta-analysis of Observational Studies in Epidemiology (MOOSE) reporting guidelines.

**Main Outcomes and Measures:**

Findings were categorized by surgical status (conservatively managed, presurgery, postsurgery, or combined); domain (eg, general cognition); type of cognitive, behavioral, or psychological measure (objective or subjective); and source of comparison data (peers or normative data).

**Results:**

Data from 32 studies, involving a pooled sample of 1422 children and adults with SS (mean [SD] age at assessment, 5.7 [6.6] years; median [interquartile range] age, 3.3 [0.5-10.3] years), were analyzed. Data on sex were available for 824 participants, and 642 (78%) were male. Individual study results varied substantially. Objective tests identified significant moderate group differences on 3 of 16 examined domains: presurgical motor functioning (3 studies; *g_w_* = −0.42; 95% CI, −0.67 to −0.18; *P* < .001), postsurgical short-term memory (2 studies; *g_w_* = −0.45; 95% CI, −0.72 to −0.17; *P* < .001), and postsurgical visuospatial ability (6 studies; *g_w_* = 0.31; 95% CI, 0.18 to 0.44; *P* < .001). Prevalence estimates and ORs varied widely, with 15 studies showing prevalence estimates ranging from 3% to 37%, and 3 studies showing ORs ranging from 0.31 (95% CI, 0.01 to 6.12) for processing speed in the conservatively managed sample to 4.55 (95% CI, 0.21 to 98.63) for postsurgical visuospatial abilities.

**Conclusions and Relevance:**

In this meta-analysis, findings for the functioning of participants with SS were highly disparate and often of low quality, with small samples sizes and control groups rarely recruited. Nonetheless, the findings suggest that some individuals with SS experience negative outcomes, necessitating routine assessment.

## Key Points

**Question:**

Do individuals with sagittal synostosis experience greater cognitive, behavioral, and/or psychological difficulties, compared with their healthy peers?

**Findings:**

In this meta-analysis, data from 32 independent studies involving a pooled sample of 1422 children and adults and examining 16 domains were analyzed. Overall, results were highly variable, with individual study results ranging from moderately positive findings for global development, where the children with sagittal synostosis were functioning at better levels than their peers, to large negative differences between groups for general cognition.

**Meaning:**

These findings suggest that some children with sagittal synostosis experience negative outcomes; thus, ongoing monitoring and referral to support services as required are critical.

## Teaser

This meta-analysis reviews research on individuals with sagittal synostosis to determine whether, and to what extent, they experience cognitive, behavioral, and psychological difficulties compared with their healthy peers or normative data for each measure.

## Introduction

Sagittal synostosis (SS), also known as scaphocephaly, occurs when the fibrous connective tissue joint that runs along the top of the skull between the 2 parietal bones (sagittal suture) fuses prematurely (ie, before adulthood), thereby restricting normal transverse growth of the skull.^1^ Instead, as the brain continues to grow, the skull compensates by growing at the remaining open cranial sutures, resulting in a long and narrow head shape with fullness (bossing) of the forehead.^2^

Of the major cranial sutures (sagittal, metopic, coronal, and lambdoid), SS is the most common craniosynostosis, found in approximately one-half of nonsyndromic (isolated) cases, occurring in approximately 2 to 3 births per 10 000, and diagnosed more frequently in boys than girls.^3^ SS cases are increasing globally, although the reasons for this increase are unknown. Suggested causal mechanisms^4,5^ include genetics (eg, *AXIN2* gene variation), gestational exposure to environmental factors (eg, maternal substance use), hormonal influences (eg, maternal thyroid dysfunction), mechanical forces (eg, intrauterine constraint), and familial cases (approximately 2% of SS cases).^6,7,8,9,10^

SS typically requires surgical management to improve the child’s appearance and/or ensure the shape of the cranial vault so that the brain can grow and develop normally. The brain growth curve guides the appropriate choice of surgical technique and timing.^11,12^ Despite these treatment aims, findings on cognitive functioning in individuals with SS indicate considerable problems. For example, general cognitive problems have been estimated to affect 4% to 37% of children with SS,^13,14,15,16^ whereas measures of general cognition have shown those with SS to be performing better than,^17^ worse than,^18^ and even comparable to^19,20^ their peers. Results for specific cognitive domains include verbal or language problems reported in 7% to 37%^14,21,22,23^ of children with SS and visuospatial deficits in 7% of children with SS.^21,24^ Parental reports indicate that behavioral problems are common, with 26% of children with SS exhibiting externalizing traits (eg, restlessness, temper tantrums) and 14% with internalizing behaviors (eg, fears).^25^ Moreover, children’s concerns with their appearance may lead to social isolation and anxiety, which can affect their psychological well-being, although research in this area is sparse.^26,27,28,29^

Interpretation of the aforementioned findings is, however, complicated by the use of diverse outcome measures (eg, Bayley Scales of Infant Development [BSID] and Wechsler Preschool and Primary Scale of Intelligence),^30,31,32,33^ in addition to differences in the surgical status of samples (eg, conservatively managed vs presurgical vs postsurgical). Moreover, pathological differences in the way that different sutures fuse—with midline sutures (ie, sagittal and metopic) being more vulnerable to some genetic variations (eg, *SMAD6*) than coronal and lambdoid sutures—could lead to differential outcomes.^3,34^ These findings highlight a need to consider the effect of distinct methodological and clinical differences when examining cognitive, behavioral, and psychological outcomes in individuals with SS.

Given the aforementioned issues, it is currently difficult to draw conclusions about the associations of SS with performance and functioning. For this reason, the current meta-analysis had 2 primary aims: to provide a comprehensive up-to-date review of this literature by identifying all research that examined cognitive, behavioral, and psychological outcomes of individuals with SS; and to determine whether and to what extent children and adults with SS experience difficulties across these domains.

## Methods

### Literature Search and Inclusion Criteria

This study follows the Meta-analysis of Observational Studies in Epidemiology (MOOSE) reporting guidelines.^35^ The review protocol was preregistered with the PROSPERO International Register of Systematic Reviews. With the assistance of an expert research librarian, comprehensive searches of the PubMed, Scopus, Embase, and PsycINFO electronic databases were conducted in August 2020 and again in January 2021, with no date restrictions (eTable 1 in the Supplement). In addition, reference lists were manually examined and Scopus citation searches undertaken for all included studies.

All studies had to meet the following criteria: (1) participants received a diagnosis of single-suture (isolated or nonsyndromic) SS or scaphocephaly; (2) cognitive, behavioral, and psychological outcomes were assessed; (3) quantitative data were suitable for the calculation of effect sizes; (4) details of the specific measure used were provided so that normative data could be obtained (where no comparison group was recruited); (5) the study was published in a peer-reviewed journal (so-called gray literature was excluded) in English; and (6) studies were original research with 2 or more participants (excludes reviews and case studies). Cases of syndromic SS (eg, Apert syndrome), multiple affected sutures, and other conditions known to affect functioning (>10% of the sample) were excluded. Studies that included participants who were identified for inclusion because they had cognitive or behavioral problems were excluded.

The literature search initially identified 12 920 records, which were imported into Covidence screening software (Veritas Health Innovation). Two authors (A.J.O. and R.M.R.) independently assessed all studies for which eligibility was ambiguous, after which a consensus decision was made. eTable 2 in the Supplement provides details for the final sample of 52 included articles (32 independent studies). Studies using overlapping samples were combined and treated as nonindependent studies (eTable 3 in the Supplement). The Figure details the study selection process.

**Figure.**
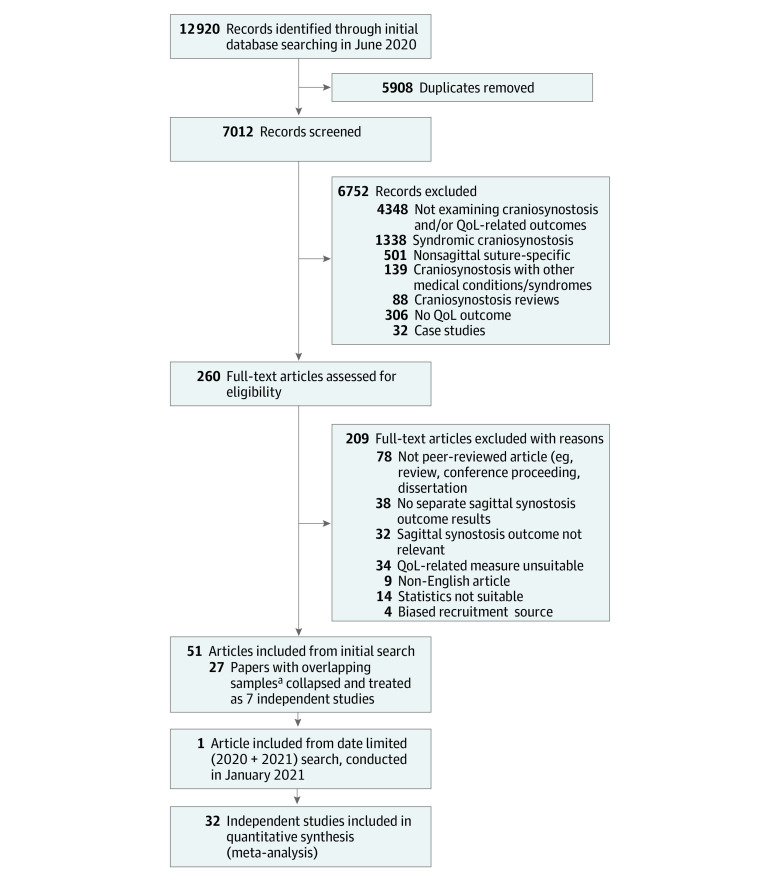
Flowchart of the Study Selection Process QoL indicates quality of life. ^a^Details of articles with overlapping samples that were collapsed and treated as independent studies are provided in eTable 3 in the Supplement.

### Data Collection and Preparation

Date extraction was performed by 2 independent reviewers (A.J.O. and a research assistant) using a prepiloted data form, focusing on demographic, clinical, methodological, and effect size data (eTable 4 in the Supplement). Discrepancies were resolved by consensus (91% agreement obtained). A total of 57 individual measures, broadly classified into 16 corresponding domains, were used to assess outcomes (eTable 5 in the Supplement).^36,37^ Individual study results were also grouped according to surgery status (ie, conservatively managed, presurgery, postsurgery, and mixed), type of measure (objective vs subjective), and comparison group (ie, healthy peers vs normative data). In addition, the impact of mean age at assessment was examined by grouping studies according to the broad developmental stage of their sample. The data were standardized before analysis, and the authors of 4 studies^19,38,39,40^ were contacted and asked to provide further information, with 1 responding.

### Study Reporting Quality

All studies were rated using modified versions of the National Institutes of Health Study Quality Assessment Tool–Observational Cohort and Cross-Sectional Studies (12 criteria) and Case-Control Studies (14 criteria). Each criterion was categorized (met, not met, not reported, or not applicable), and the percentage of included studies that met each criterion was determined. Data extraction was undertaken by 2 independent coders (A.J.O. and a research assistant), and discrepancies were resolved by consensus.

### Statistical Analysis

Effect size data were entered into Comprehensive Meta-Analysis statistical software version 3.3 (Biostat, Inc) with conservative random-effects models generated. *P* values assessed statistical significance (set at 2-sided *P* < .05), and 95% CIs determined precision.

Three effect sizes were calculated. First, standardized mean differences (Hedges *g*) compared individuals with SS vs healthy control participants or normative data (24 studies). Effect sizes were weighted (inverse variance method) and pooled (*g_w_*). A negative* g_w_* indicated poorer functioning in the SS group vs the comparisons, whereas a positive* g_w_* indicated better functioning.^41^ Second, proportions, weighted by sample size, examined the prevalence of cognitive, behavioral, and psychological problems in those with SS (15 studies). Third, odds ratios (ORs) determined the likelihood (increased occurrence, OR > 1; decreased occurrence, OR < 1) of experiencing cognitive, behavioral, or psychological problems after SS compared with healthy peers (3 studies).

Between-study heterogeneity was explored using prediction intervals (pooled analyses with ≥5 included studies), which represent the extent to which the true effect size varies across populations,^42^ in addition to *I*^2^ and τ values (≥2 included studies). Orwin failsafe *N* (*N*_fs_) values were calculated for pooled analyses with 2 or more studies, given that the small number of studies in each analysis (range, 1-9 studies) rendered more formal statistical tests of publication bias problematic.

## Results

Summary details for the 32 independent studies (1422 participants) included in the meta-analysis are shown in Table 1, with study-specific information provided in eTable 2 in the Supplement. The majority of studies included small sample sizes (mean [SD], 44.4 [42.5] participants) and children (mean [SD] age at assessment, 5.7 [6.6] years; median [interquartile range], 3.3 [0.5-10.3] years). Data on sex were available for 824 participants, and 642 (78%) were male. Samples were typically assessed postsurgically (25 studies), and comparison groups were not routinely used (23 studies).

**Table 1.** Summary Details for the 32 Independent Studies Included in the Meta-analysis

Characteristic	Studies, No.^a^	Participants, No. (%)^a^	Value
Sample size, mean (SD), No. of participants	32	1422	44.4 (42.5)
Age at first assessment, y			
Mean (SD)	24	1208	5.7 (6.6)
Median (interquartile range)			3.3 (0.5-10.3)
Surgical status, mean (SD), No. of participants^b^			
CM	4	98	24.5 (20.7)
Presurgery	13	581	44.7 (38.7)
Postsurgery	25	915	36.6 (32.4)
Mixed^c^	4	193	48.3 (32.5)
Sex^d^			
Female	20	182 (22.0)	NA
Male	20	642 (78.0)	NA
Study focus			
Studies examining sagittal synostosis only	18	996 (70.0)	NA
Studies examining multiple suture types	14	426 (30.0)	NA
Control group used			
Yes	9	262 (18.4)	NA
No	23	1160 (81.6)	NA
Origin of study			
US	15	469 (32.9)	NA
Europe	10	534 (37.6)	NA
United Kingdom	3	283 (19.9)	NA
Asia	2	104 (7.3)	NA
Australia	2	32 (2.3)	NA
Surgical technique reported			
Yes	16	801 (56.3)	NA
No	12	459 (32.3)	NA
CM or presurgery only	4	162 (11.4)	NA
Surgical procedures, No.			
1	3	48 (3.4)	NA
≥2	6	216 (15.2)	NA
Not reported	20	1042 (73.3)	NA
CM or presurgery only	3	116 (8.1)	NA
Criteria for surgery reported			
Yes	6	170 (12.0)	NA
No	26	1252 (88.0)	NA
Family history of craniosynostosis reported			
Yes	0	0	NA
No	32	1422 (100.0)	NA
Genetic status examined			
Yes	5	373 (26.2)	NA
No	27	1049 (73.8)	NA
Socioeconomic status examined			
Yes	10	496 (34.9)	NA
No	22	926 (65.1)	NA
Neonatal and/or perinatal problems reported			
Yes	9	483 (34.0)	NA
No	23	939 (66.0)	NA

Abbreviations: CM, conservatively managed; NA, not applicable.

^a^ Refers to the total number of studies and participants for which data were available.

^b^ Some studies reported data for multiple groups.

^c^ Refers to CM, presurgery, and postsurgery combined.

^d^ Data on sex were available for 824 participants.

Overall reporting quality for each of the included studies was poor. Results for single-group observational studies are presented in eTable 6 and eFigure 1 in the Supplement, whereas findings for case-control studies are reported in eTable 7 and eFigure 2 in the Supplement.

Estimated effect sizes varied considerably across the included studies, as reflected by the heterogeneity indices (Table 2, Table 3, and Table 4 and eTable 8 in the Supplement). The results, for the most part, were also susceptible to publication bias (*N*_fs_). Individual findings, grouped by surgical status (conservatively managed, presurgery, postsurgery, and combined) are discussed in more detail in the following subsections.

**Table 2.** Outcomes of Children and Adults With Conservatively Managed SS

Domain and comparison data	Standardized mean group differences							Prevalence rates					
	Studies, No.	Participants with SS, No.	Hedges *g* (95% CI)	*P* value	*I* ^2^	τ	*N*_fs_	Studies, No.	Participants with SS, No.	Prevalence (95% CI)	Studies, No.	Participants with SS, No.	OR (95% CI)^a^
Objective measures													
General cognition													
Peers	1	30	−0.08 (−0.67 to 0.51)	.79	NA	NA	NA	2	65	0.05 (0.02 to 0.14)	NA	NA	NA
Norms^b^	2	17	0.19 (−0.29 to 0.67)	.43	0.00	0.00	0						
Motor functioning, norms	1	13	−0.15 (−0.70 to 0.39)	.59	NA	NA	NA	NA	NA	NA	NA	NA	NA
Verbal abilities, norms	1	14	0.09 (−0.44 to 0.63)	.74	NA	NA	NA	1	14	0.07 (0.01 to 0.37)	1	13	0.33 (0.02 to 6.65)
Visuospatial abilities, norms	1	14	0.02 (−0.51 to 0.56)	.93	NA	NA	NA	1	17	0.07 (0.01 to 0.36)	1	16	0.69 (0.04 to 11.11)
Adaptive and social skills, norms	1	13	−0.14 (−0.69 to 0.41)	.62	NA	NA	NA	NA	NA	NA	NA	NA	NA
Arithmetic	NA	NA	NA	NA	NA	NA	NA	NA	NA	NA	1	18	0.71 (0.04 to 12.43)
Attention	NA	NA	NA	NA	NA	NA	NA	1	19	0.13 (0.03 to 0.41)	1	26	1.04 (0.48 to 2.27)
Executive function	NA	NA	NA	NA	NA	NA	NA	1	12	0.08 (0.01 to 0.41)	NA	NA	NA
Global development, norms	1	23	0.58 (0.17 to 0.99)	.01	NA	NA	NA	NA	NA	NA	NA	NA	NA
Memory: shorter-term	NA	NA	NA	NA	NA	NA	NA	1	18	0.09 (0.03 to 0.23)	1	16	0.70 (0.05 to 10.33)
Processing speed	NA	NA	NA	NA	NA	NA	NA	1	14	0.07 (0.01 to 0.37)	1	14	0.31 (0.01 to 6.12)
Subjective measures													
Behavior													
Externalizing, norms	1	24	−0.25 (−0.65 to 0.15)	.22	NA	NA	NA	NA	NA	NA	NA	NA	NA
Internalizing, norms	1	24	−0.30 (−0.70 to 0.10)	.14	NA	NA	NA	NA	NA	NA	NA	NA	NA
Overall, norms	1	24	−0.29 (−0.69 to 0.11)	.16	NA	NA	NA	NA	NA	NA	NA	NA	NA
Quality of life, norms	1	24	0.00 (−0.40 to 0.40)	>.99	NA	NA	NA	NA	NA	NA	NA	NA	NA

Abbreviations: NA, not applicable; *N*_fs_, Orwin failsafe *N*; OR, odds ratio; SS, sagittal synostosis.

^a^ ORs are shown on a logarithmic scale.

^b^ Norms refers to comparisons against normative data for each measure.

**Table 3.** Outcomes of Children With SS Who Had Not Yet Undergone Surgery

Domain and comparison data	Standardized mean group differences							Prevalence rates		
	Studies, No.	Participants with SS, No.	Hedges *g* (95% CI)	*P* value	*I* ^2^	τ	*N*_fs_	Studies, No.	Participants with SS, No.	Prevalence (95% CI)
General cognition										
Peers	3	89	−0.28 (−0.67 to 0.10)	.15	34.48	0.21	1	1	27	0.37 (0.21 to 0.56)
Norms^a^	4	160	−0.15 (−0.89 to 0.60)	.70	94.99	0.74	0			
Motor functioning										
Peers	3	108	−0.42 (−0.67 to −0.18)	<.001	0.00	0.00	3	2	114	0.17 (0.07 to 0.36)
Norms	4	166	−0.30 (−10.01 to 0.41)	.41	94.80	0.71	2			
Verbal abilities										
Peers	3	99	0.01 (−0.24 to 0.26)	.96	0.00	0.00	0	2	90	0.13 (0.03 to 0.40)
Norms	2	103	0.17 (−0.03 to 0.37)	.09	0.00	0.00	0			
Visuospatial abilities										
Peers	1	28	−0.09 (−0.61 to 0.43)	.73	NA	NA	NA	1	87	0.07 (0.03 to 0.15)
Norms	1	26	0.44 (0.04 to 0.83)	.03	NA	NA	NA			
Adaptive and social skills										
Peers	1	28	−0.21 (−0.72 to 0.31)	.44	NA	NA	NA	1	87	0.06 (0.02 to 0.13)
Norms	1	26	0.21 (−0.60 to 0.18)	.30	NA	NA	NA			
Global development										
Peers	1	28	−0.31 (−0.83 to 0.21)	.25	NA	NA	NA	5	378	0.15 (0.09 to 0.24)
Norms	1	26	0.30 (−0.09 to 0.70)	.13	NA	NA	NA			

Abbreviations: NA, not applicable; *N*_fs_, Orwin failsafe *N*; SS, sagittal synostosis.

^a^ Norms refers to comparisons against normative data for each measure.

**Table 4.** Outcomes of Children and Adults With SS Who Had Undergone Surgery

Domain and comparison data	Standardized mean group differences							Prevalence rates					
	Studies, No.	Participants with SS, No.	Hedges *g* (95% CI)	*P* value	*I* ^2^	τ	*N*_fs_	Studies, No.	Participants with SS, No.	Prevalence (95% CI)	Studies, No.	Participants with SS, No.	OR (95% CI)^a^
Objective measures													
General cognition													
Peers	2	113	−0.12 (−0.57 to 0.33)	.60	49.78	0.24	0	3	112	0.20 (0.11 to 0.34)	NA	NA	NA
Norms^b^	9	299	0.01 (−0.26 to 0.27)	.96	75.71	0.34	0						
Motor functioning													
Peers	3	131	0.18 (−0.41 to 0.05)	.13	0.00	0.00	0	2	81	0.12 (0.03 to 0.36)	NA	NA	NA
Norms	7	244	0.01 (−0.31 to 0.34)	.93	83.10	0.40	0						
Verbal abilities													
Peers	3	122	−0.65 (−1.34 to 0.03)	.06	74.77	0.52	7	3	84	0.15 (0.08 to 0.27)	NA	NA	NA
Norms	7	266	0.23 (−0.01 to 0.47)	.06	69.71	0.26	1						
Visuospatial abilities													
Peers	3	102	−0.07 (−0.32 to 0.17)	.56	0.00	0.00	0	1	63	0.05 (0.02 to 0.14)	1	35	4.55 (0.21 to 98.63)
Norms	6	248	0.31 (0.18 to 0.44)	<.001	0.00	0.00	3						
Adaptive and social skills, norms	2	76	0.07 (−0.26 to 0.41)	.67	48.57	0.17	0	1	63	0.03 (0.01 to 0.12)	NA	NA	NA
Arithmetic													
Peers	1	75	−0.21 (−0.48 to 0.06)	.12	NA	NA	NA	NA	NA	NA	NA	NA	NA
Norms	1	10	−0.24 (−0.86 to 0.38)	.45	NA	NA	NA	NA	NA	NA	NA	NA	NA
Attention													
Peers	2	94	−0.40 (−1.08 to 0.29)	.26	73.58	0.43	2	NA	NA	NA	NA	NA	NA
Norms	1	38	−0.51 (−0.84 to −0.19)	<.001	NA	NA	NA	NA	NA	NA	NA	NA	NA
Executive function, peers	1	75	−0.18 (−0.45 to 0.09)	.19	NA	NA	NA	NA	NA	NA	NA	NA	NA
Global development, norms	2	76	0.34 (−0.09 to 0.76)	.13	68.17	0.26	1	1	26	0.15 (0.06 to 0.35)	NA	NA	NA
Learning difficulties	NA	NA	NA	NA	NA	NA	NA	4	143	0.23 (0.11 to 0.41)	1	70	0.64 (0.35 to 1.17)
Memory													
Shorter term													
Peers	2	92	−0.20 (−0.45 to 0.05)	.12	<.001	0.00	0	NA	NA	NA	NA	NA	NA
Norms	2	51	−0.45 (−0.72 to −0.17)	<.001	<.001	0.00	3	NA	NA	NA	NA	NA	NA
Longer term, peers	1	74	−0.18 (−0.45 to 0.10)	.20	NA	NA	NA	NA	NA	NA	NA	NA	NA
Processing speed, norms	2	48	−0.26 (−1.14 to 0.63)	.57	79.89	0.58	1	NA	NA	NA	NA	NA	NA
Subjective measures													
Motor functioning	NA	NA	NA	NA	NA	NA	NA	1	138	0.10	NA	NA	NA
Verbal abilities, peers	1	70	−0.01 (−0.31 to 0.28)	.93	NA	NA	NA	1	138	0.09	NA	NA	NA
Adaptive and social skills, norms	1	75	0.15 (−0.08 to 0.38)	.19	NA	NA	NA	1	140	0.12	NA	NA	NA
Attention, peers	1	76	−0.02 (−0.31 to 0.26)	.87	NA	NA	NA	NA	NA	NA	NA	NA	NA
Behavior													
Externalizing													
Peers	2	83	−0.04 (−0.30 to 0.22)	.76	0.00	0.00	0	1	94	0.26 (0.20 to 0.32)	1	76	1.48 (0.79 to 2.74)
Norms	3	192	−0.14 (−0.29 to 0.02)	.08	13.45	0.05	0						
Internalizing													
Peers	2	83	−0.19 (−0.62 to 0.25)	.40	24.24	.20	0	1	94	0.14 (0.10 to 0.20)	1	76	1.17 (0.65 to 2.09)
Norms	3	192	−0.07 (−0.21 to 0.07)	.32	0.00	0.00	0						
Overall													
Peers	2	83	0.02 (−0.33 to 0.38)	.91	13.13	0.14	0	1	94	0.22 (0.15 to 0.32)	1	76	1.52 (0.83 to 2.81)
Norms	1	75	0.17 (−0.15 to 0.49)	.30	NA	NA	NA						
Executive function													
Peers	1	75	0.04 (−0.25 to 0.33)	.79	NA	NA	NA	NA	NA	NA	NA	NA	NA
Norms	3	192	−0.40 (−0.66 to −0.14)	<.001	68.20	0.19	3	NA	NA	NA	NA	NA	NA
Memory: shorter term, norms	3	192	−0.39 (−0.63 to −0.15)	<.001	62.22	0.17	3	NA	NA	NA	NA	NA	NA
Quality of life, norms	1	5	1.29 (0.42 to 2.17)	<.001	NA	NA	NA	NA	NA	NA	NA	NA	NA
Satisfaction with appearance, peers	1	40	−0.06 (−0.50 to 0.37)	.78	NA	NA	NA	NA	NA	NA	NA	NA	NA
Self-concept, peers	1	7	−0.03 (−0.95 to 0.88)	.94	NA	NA	NA	NA	NA	NA	NA	NA	NA

Abbreviations: NA, not applicable; *N*_fs_, Orwin failsafe *N*; OR, odds ratio; SS, sagittal synostosis.

^a^ ORs are shown on a logarithmic scale.

^b^ Norms refers to comparisons against normative data for each measure.

### Conservatively Managed Samples

Four independent studies (9 articles)^16,21,43,44,45,46,47,48^ examined 13 domains (Table 2 and eFigure 3 in the Supplement). Only 1 domain, global development, reached significance: the SS group functioned better than published normative data in 1 study (*g* = 0.58; 95% CI, 0.17 to 0.99; *P* = .01).^43^ Estimates provided by individual studies in this domain were, however, imprecise, as reflected by the wide 95% CIs. The single study that recruited healthy peers found a small but nonsignificant negative effect size for general cognition (*g* = −0.08; 95% CI, −0.67 to 0.51; *P* = .79),^21^ whereas the remaining 2 studies^43,47^ contributing to this domain reported small positive effect sizes. Similarly, the single study that used parent ratings of child behavior and quality of life reported no significant group differences, despite parents rating the behavior of their child with SS more poorly than their sibling.^21^

Prevalence estimates of cognitive difficulties among SS groups were modest, ranging from 5% (general cognition) to 13% (attention). With regard to ORs, no domains reached significance, but the SS group functioned better across some domains, as suggested by OR values ranging from 0.31 (95% CI, 0.01-6.12) for processing speed to 0.71 (95% CI, 0.04-12.43) for arithmetic to 4.55 (95% CI, 0.21-98.63) for visuospatial abilities. The nonsignificant findings may reflect the small SS sample sizes (<26 participants) used in these studies.

### Presurgical Samples

Presurgical outcomes are based on 13 independent studies (21 articles)^13,15,16,18,20,23,24,43,44,45,46,47,48,49,50,51,52,53,54,55,56^ (Table 3 and eFigure 4 in the Supplement). Mean pooled group differences were typically larger compared with healthy peers, although the differences were not significant (eg, general cognition: *g*_w_ for peers = −0.28; 95% CI, −0.67 to 0.10; *P* = .15; *g*_w_ for normative data = −0.15; 95% CI, −0.89 to 0.60; *P* = .70). Only tests of motor functioning reached significance (*g*_w_ = −0.42; 95% CI, −0.67 to −0.18; *P* < .001). One notable individual finding (eFigure 4 in the Supplement) was a study demonstrating very large and significant mean differences in both general cognition (*g*_w_ = −1.03; 95% CI, −1.36 to −0.71; *P* < .001) and motor functioning (*g*_w_ = −1.04; 95% CI, −1.37 to −0.71; *P* < .001) indicating that, before surgery, these children were functioning a full SD below BSID second version norms.^15,54^

Up to 37% of children (10 of 27 children) were identified as experiencing cognitive impairment, with 6% (5 of 87 children) demonstrating problems with adaptive and social functioning (eg, waving bye-bye). Despite individual study differences, with small sample size studies likely providing less reliable data, pooled and weighted prevalence estimates were comparable across domains (range, 13% to 17%).

### Postsurgical Samples

Among the 25 independent studies (42 articles)^15,16,18,19,20,22,23,24,25,38,43,44,45,46,47,48,49,54,55,57,58,59,60,61,62,63,64,65,66,67,68,69,70,71,72,73,74,75,76,77,78,79^ examining postsurgical results, the largest effect sizes were seen among those that compared test scores of SS groups with those of healthy peers, indicating poorer performances among the former (Table 4 and eFigure 5 in the Supplement). However, only studies that involved normative data comparisons reached significance: moderate group differences were noted for visuospatial abilities (6 studies; *g_w_* = 0.31; 95% CI, 0.18 to 0.44; *P* < .001), attention (1 study; *g_w_* = −0.51; 95% CI, −0.84 to −0.19; *P* < .001), and shorter-term memory (2 studies; *g_w_* = −0.45; 95% CI, −0.72 to −0.17; *P* < .001). The diversity of results is reflected among studies that assessed verbal abilities, specifically, with individual studies (eFigure 5 in the Supplement) in this domain reporting moderate-to-large and negative effects (peer comparison) but also a small positive effect (normative data comparison).

Where subjective measures were used (8 studies), significant group differences in executive functioning (3 studies; *g_w_* = −0.40; 95% CI, −0.66 to −0.14; *P* < .001), short-term memory (3 studies *g_w_* = −0.39; 95% CI, −0.63 to −0.15; *P* < .001), and quality of life (1 study; *g_w_* = 1.29; 95% CI, 0.42 to 2.17; *P* < .001) were noted: parents reported poorer cognition, but also enhanced quality of life, for their child compared with normative data. These findings should be considered tentative, given the small number of studies that contributed to these data.

Prevalence rates based on objectively assessed problems (9 studies) identified significant learning difficulties (33 of 143 children [23%]) and general cognitive problems (22 of 112 children [20%]) among children with SS, with fewer problems reported in other domains. In addition, parents and teachers reported a higher rate of externalizing behavior problems (24 of 94 children [26%]) compared with internalizing behavior problems (13 of 94 children [14%]), whereas few problems with adaptive and social functioning were reported (2 of 63 children [3%]).

### Surgical Status Not Specified

The data for 4 independent studies (7 articles)^43,44,45,46,80,81^ examining mixed SS samples were compared with normative data (eTable 9 in the Supplement). Of the 4 domains examined, only motor functioning was associated with a significant group difference: the SS group displayed poorer function in comparison to normative data (1 study; *g* = −0.93; 95% CI, −1.18 to −0.69; *P* < .001). Moreover, a large percentage of children were found to have verbal (28 of 76 children [37%]) and general cognitive (14 of 71 children [20%]) issues.

### Age at Assessment

Findings were analyzed according to mean age at assessment (24 studies). However, there was no clear pattern of findings (eFigure 6 in the Supplement).

## Discussion

Data from 32 independent studies, involving a pooled sample of 1422 children and adults, were analyzed. Overall, results were highly variable, ranging from individual study results including moderate positive findings, whereby children with SS were functioning at better levels than their peers, to large negative differences.

Few studies examined the outcomes of children whose SS was conservatively managed. Only 1 study^21^ recruited controls (siblings), thereby controlling for family-level environmental factors; however, siblings of children with health conditions are also known to experience difficulties, limiting the conclusions that can be drawn.^82,83^ Global development was the only domain to demonstrate that participants with SS were performing better than the normative data; however, the Griffiths Mental Development Scale (GMDS)^44^ has been shown to overestimate developmental functioning.^84,85,86^ Moreover, normative data for the GMDS were not updated for some years; thus, higher test scores may reflect the Flynn effect, whereby raw intelligence quotient scores increase over time.^87^ Siblings were also used as controls in the single study that assessed behavior and quality of life, using parental reports to compare their children (ie, child with SS vs sibling).^21^ No differences were found between groups, perhaps highlighting the challenges of using siblings and, moreover, the importance of multi-informant ratings to mitigate differential effects of child and parent ratings.

Findings on cognitive functioning for children in the presurgical sample who later went on to have surgery were also highly variable, indicating that although some children with SS experience cognitive difficulties, this is not always the case. It is noteworthy that, on all assessed domains, the SS group performed more poorly when compared with healthy controls rather than normative data, although few of these group differences were significant. Notably, different versions of the same measure, such as the BSID (first, second, and third versions), generated different effect estimates, ranging from moderate negative to moderate positive group differences. These findings suggest that factors other than surgical status and the measure used (eg, socioeconomic status) need to be considered. Similar to the GMDS, the BSID third version may also overestimate child development^88^; hence, the findings may reflect an artifact of the actual measure used, rather than an accurate indication of child development.

Findings for children who had undergone surgical treatment for their SS were also highly disparate. Notably, subjective ratings by parents on the Brief Working Memory Index reflected those on objective tests (eg, Children’s Memory Scale), suggesting that parents were accurately observing and reporting behavioral signs of their child’s short-term memory difficulties. No significant group differences in psychological functioning were reported; hence, the experience of having SS and its associated surgical procedures in early childhood may play a lifelong role in increasing people’s resilience and outlook.^78^ Qualitative studies examining the psychological impact of SS on the individual and their families may provide greater detail about the strengths imparted to all family members during these challenges.

The understanding of the SS disease process has evolved, and, with the advent of surgical innovations, the operative approaches for managing patients have changed considerably, from early suturectomy to more extensive cranial vault remodeling and variants thereof.^11^ More recently, spring-assisted surgery and endoscopic-assisted craniectomies with or without helmets have been described.^1,77^ There is a wide disparity of opinions about the appropriate treatment of SS, with extended calvarial vault remodeling being the most commonly performed procedure worldwide.^89^ This technique is reproducible and adheres to the principle of removing the affected suture, spanning the adjacent functional suture, and, thus, reducing the primary deformity and allowing space for the brain to grow and expand unhindered.^11^ Similarly, there is no consensus on the optimal age for surgery, with the brain growth curve influencing both the appropriate choice of technique and timing.^12^ Both these factors are likely be associated with outcomes, with additional research needed to clarify the impact of both the short-term and long-term functioning of children and adults with SS who have undergone surgery.

### Limitations

There are a number of limitations that warrant consideration. First, because comparison groups were often not recruited, normative data from the specific measure were used to generate effect sizes. Normative data may not be representative because of changes in population composition (eg, education, age, or economic status) over time,^90^ highlighting a need for future research to recruit appropriately matched comparison groups. Second, potentially relevant studies were excluded because they did not specify which version of a measure was used, and/or normative data could not be accessed. Similarly, some data could not be used because studies combined their findings for different sutures (eg, sagittal and metopic), despite known pathophysiological differences between sutures.^34^ Future research should report data for different sutures separately. Third, the exact criteria used to identify children with problems differed between studies and, moreover, the measures used to assess neurodevelopmental functioning in infants (eg, GMDS) may not accurately predict later performance.^91,92^ Fourth, studies did not consistently report key sample parameters, such as age, sex, and type of surgical treatment, which are variables that should be examined because they have been shown to be associated with cognitive functioning.^46,69^

## Conclusions

The current findings highlight that, on the basis of the reviewed literature, there were no consistent associations between SS and neurocognitive delays. Nonetheless, some children were experiencing difficulties (eg, attention or short-term memory problems), indicating that ongoing monitoring and assessment are important to ensure that children with difficulties are referred to support services as required. Future research should recruit larger samples with well-matched comparison groups and should examine conservatively managed samples more often, in addition to comprehensively reporting both sample characteristics and outcomes, according to important demographic (eg, age and sex) and clinical (eg, surgical technique) variables.

## Notes

Supplement. **eTable 1.** Electronic Search Strategy **eTable 2.** Summary Details of the Meta-analyzed Studies **eTable 3.** Studies Using Overlapping Samples - Combined and Treated as Non-independent Studies in the Current Meta-analysis **eTable 4.** Data Extracted From Studies Included in the Meta-analysis (Where Reported) **eTable 5.** Summary of the Cognitive Tests That Were Used by the Studies and Their Corresponding Cognitive Domain **eTable 6.** Adapted NIH Quality Assessment of Observational Cohort and Cross-Sectional Studies **eTable 7.** NIH Quality Assessment of Case-Control Studies **eTable 8.** Prediction Intervals for Pooled Analyses With ≥5 Included Studies **eTable 9.** Outcomes of Mixed (Conservatively Managed + Presurgical + Postsurgical) Samples of Children With Sagittal Synostosis **eFigure 1.** Percentage of Observational Cross-Sectional and Cohort Studies Meeting Each of the Adapted NIH Study Quality Criteria (N_studies_ = 23) **eFigure 2.** Percentage of Case-Control Studies Meeting Each of the Adapted NIH Study Quality Criteria (N_studies_ = 9) **eFigure 3.** Outcomes of Children With Conservatively Managed Sagittal Synostosis **eFigure 4.** Presurgical Functioning of Children With Sagittal Synostosis: Had Not Yet Undergone Surgery Prior to Being Assessed **eFigure 5. **Outcomes of Children With Operated Sagittal Synostosis: Had Undergone Surgery Prior to Being Assessed **eFigure 6.** Pooled Analyses for Each Cognitive, Behavioral and Psychological Domain, Partitioned According to Surgical Status (Conservatively Managed, Presurgical, Postsurgical) and Age at Assessment: A (0 – 0.11); B (1:0 – 2:11); C (3:0 – 6:11); D (7:0 – 17:11); E (>18:0)
eReferences
