# Supplementary material for: Sagittal Synostosis and Its Association With Cognitive, Behavioral, and Psychological Functioning: A Meta-analysis
Source: JAMA Netw Open. 2021 Sep 13;4(9):e2121937. doi: 10.1001/jamanetworkopen.2021.21937 (PMC8438597; doi:10.1001/jamanetworkopen.2021.21937)
Supplement: Supplement. — eTable 1. Electronic Search Strategy eTable 2. Summary Details of the Meta-analyzed Studies eTable 3. Studies Using Overlapping Samples - Combined and Treated as Non-independent Studies in the Current Meta-analysis eTable 4. Data Extracted From Studies Included in the Meta-analysis (Where Reported) eTable 5. Summary of the Cognitive Tests That Were Used by the Studies and Their Corresponding Cognitive Domain eTable 6. Adapted NIH Quality Assessment of Observational Cohort and Cross-Sectional Studies eTable 7. NIH Quality Assessment of Case-Control Studies eTable 8. Prediction Intervals for Pooled Analyses With ≥5 Included Studies eTable 9. Outcomes of Mixed (Conservatively Managed + Presurgical + Postsurgical) Samples of Children With Sagittal Synostosis eFigure 1. Percentage of Observational Cross-Sectional and Cohort Studies Meeting Each of the Adapted NIH Study Quality Criteria (Nstudies = 23) eFigure 2. Percentage of Case-Control Studies Meeting Each of the Adapted NIH Study Quality Criteria (Nstudies = 9) eFigure 3. Outcomes of Children With Conservatively Managed Sagittal Synostosis eFigure 4. Presurgical Functioning of Children With Sagittal Synostosis: Had Not Yet Undergone Surgery Prior to Being Assessed eFigure 5. Outcomes of Children With Operated Sagittal Synostosis: Had Undergone Surgery Prior to Being Assessed eFigure 6. Pooled Analyses for Each Cognitive, Behavioral and Psychological Domain, Partitioned According to Surgical Status (Conservatively Managed, Presurgical, Postsurgical) and Age at Assessment: A (0 – 0.11); B (1:0 – 2:11); C (3:0 – 6:11); D (7:0 – 17:11); E (>18:0) eReferences [file jamanetwopen-e2121937-s001.pdf]

Supplemental Online Content

Osborn AJ, Roberts RM, Dorstyn DS, Grave BG, David DJ. Sagittal synostosis and its association with cognitive, behavioral, and psychological functioning: a meta-analysis. *JAMA Netw Open*. 2021;4(9):e2121937. doi:10.1001/jamanetworkopen.2021.21937

eTable 1. Electronic Search Strategy

eTable 2. Summary Details of the Meta-analyzed Studies

eTable 3. Studies Using Overlapping Samples - Combined and Treated as Non-independent Studies in the Current Meta-analysis

eTable 4. Data Extracted From Studies Included in the Meta-analysis (Where Reported)

eTable 5. Summary of the Cognitive Tests That Were Used by the Studies and Their Corresponding Cognitive Domain

eTable 6. Adapted NIH Quality Assessment of Observational Cohort and Cross-Sectional Studies

eTable 7. NIH Quality Assessment of Case-Control Studies

eTable 8. Prediction Intervals for Pooled Analyses With ≥5 Included Studies

eTable 9. Outcomes of Mixed (Conservatively Managed + Presurgical + Postsurgical) Samples of Children With Sagittal Synostosis

eFigure 1. Percentage of Observational Cross-Sectional and Cohort Studies Meeting Each of the Adapted NIH Study Quality Criteria ($N_{studies}$ = 23)

eFigure 2. Percentage of Case-Control Studies Meeting Each of the Adapted NIH Study Quality Criteria ($N_{studies}$ = 9)

eFigure 3. Outcomes of Children With Conservatively Managed Sagittal Synostosis

eFigure 4. Presurgical Functioning of Children With Sagittal Synostosis: Had Not Yet Undergone Surgery Prior to Being Assessed

eFigure 5. Outcomes of Children With Operated Sagittal Synostosis: Had Undergone Surgery Prior to Being Assessed

eFigure 6. Pooled Analyses for Each Cognitive, Behavioral and Psychological Domain, Partitioned According to Surgical Status (Conservatively Managed, Presurgical, Postsurgical) and Age at Assessment: A (0 – 0.11); B (1:0 – 2:11); C (3:0 – 6:11); D (7:0 – 17:11); E (>18:0)

eReferences

This supplemental material has been provided by the authors to give readers additional information about their work.

© 2021 Osborn AJ et al. *JAMA Network Open.*

eTable 1

Electronic Search Strategy

<u>Pubmed</u>

(craniosynostos*[mh] OR craniosynostos*[tw] OR sagittal[tw] OR scaphocephal*[tw])

AND

(quality of life[tw] OR satisfaction with life[tw] OR life satisfaction[tw] OR anxiety[mh] OR anxiety[tw] OR anxious[tw] OR depression[mh] OR depress*[tw] OR mood[tw] OR affect[tw] OR emotion*[tw] OR psychol*[tw] OR psychiat*[TW] OR psychosocial[tw] OR psychopath*[tw] OR body, physical appearance[mh] OR appearance[tw] OR function*[tw] OR mental processes[mh] OR mental*[tw] OR cognit*[tw] OR neuropsychol*[tw] OR learning[tw] OR neurodevelopment*[tw] OR development*[tw] OR behaviour[mh] OR behav*[tw] OR neurobehav*[tw] OR memory[tw] OR language[tw] OR verbal[tw] OR visuospatial[tw] OR attention[tw] OR information processing[tw] OR academic[tw] OR intellectual[tw] OR intelligen*[tw] OR speech[tw])

PsycINFO

(craniosynostos* or sagittal or scaphocephal*).mp AND (outcome* or quality of life or satisfaction with life or life satisfaction or anxiety or anxious or depress* or mood or affect or emotion* or psychol* or psychiat* or psychosocial or psychopath* or appearance or function* or mental* or cognit* or neuropsychol* or learning or neurodevelopment* or development* or behav* or neurobehav* or memory or language or verbal or visuospatial or attention or information processing or academic or intellectual or intelligen* or speech).mp

<u>SCOPUS</u>

(craniosynostos* OR sagittal OR scaphocephal*)

AND

(outcome* OR "quality of life" OR "satisfaction with life" OR "life satisfaction" OR anxiety OR anxious OR depress* OR mood OR affect OR emotion* OR psychol* OR psychiat* OR psychosocial OR psychopath* OR appearance OR function* OR mental* OR cognit* OR neuropsychol* OR learning OR neurodevelopment* OR development* OR behav* OR neurobehav* OR memory OR language OR verbal OR visuospatial OR attention OR "information processing" OR academic OR intellectual OR intelligen* OR speech)

© 2021 Osborn AJ et al. *JAMA Network Open.*

Embase

'craniofacial synostosis'/syn OR craniosynostos* OR sagittal OR scaphocephal*

AND

outcome OR 'quality of life'/syn OR 'satisfaction with life' OR 'life satisfaction' OR 'mental disease assessment'/syn OR 'mental disease'/syn OR 'mental function assessment'/syn OR 'physical appearance'/syn OR 'cognition'/syn OR 'individual behavior assessment'/syn OR 'behavior'/syn OR 'cognitive function test'/syn OR 'psychological and psychiatric procedures'/syn OR 'learning'/syn OR 'neuropsychology'/syn OR 'memory'/syn OR 'speech and language assessment'/syn OR 'academic achievement'/syn OR neurodevelopment* OR development* OR anxiety OR anxious OR depress* OR psychol* OR psychiat* OR psychosocial OR psychopath* OR appearance OR function* OR mood OR affect OR emotion OR mental* OR cognit* OR neuropsychol* OR learning OR behav* OR neurobeh* OR memory OR language OR verbal OR visuospatial OR attention OR intellect* OR intelligen* OR speech

© 2021 Osborn AJ et al. *JAMA Network Open.*

eTable 2

Summary Details of the Meta-analyzed Studies

					**Surgical status of patients**				**Craniosynostosis risks reported**		**Cognitive risks reported**		**Outcome: domain**
Author (year)	*N*	*Mean age (yrs)*	*% male*	*Study origin*	*CM*	*pre*	*post*	*mixed*	*Genetic status*	*Family history*	*SES*	*Neonatal /perinatal*	
Arnaud et al., 1995[1]	193	T1: 1.0<br>T2: -	-	France	51	142	47						general cognition, global development
Bellew et al., 2005[2]<br>Bellew et al., 2011[3]<br>Bellew et al., 2015[4]<br>Bellew et al., 2019[5]	67	Surgical:<br>T1: 0.6<br>T2: 1.3<br>CM:<br>T1: 2.1<br>T2: 2.9	83	UK	13	28	54	47				✓	general cognition, motor functioning, verbal, visuospatial, adaptive & social skills, global development
Boltshauser et al., 2003[6]	30	9.3	77	Switzerland	30						✓		general cognition, verbal, visuospatial, arithmetic, attention, executive function, memory: shorter term, processing speed, quality of life, behavior: externalizing/internalizing/total
Byun et al., 2018[7]	67	-	69	Sth Korea				67	✓				general cognition, motor functioning
Cabrejo et al., 2019[8]	10	12.1	80	US			10						verbal, visuospatial
Care et al., 2019[9]	140	3.3	-	UK			140		✓				motor functioning, verbal, adaptive & social skills, behavior: externalizing/internalizing/total
Chandler et al., 2020[10]	75	10.3	64	US			75				✓	✓	executive function, memory-shorter term, behavior: externalizing/internalizing/total
Chieffo et al., 2010[11]	35	13.4	77	Italy			35		✓		✓		visuospatial, processing speed
Chieffo et al., 2020[12]	87	T1: 0.5<br>T2: 1.2	-	Italy		87	63						global development, motor functioning, verbal, visuospatial, adaptive & social skills
Chuang et al., 2018[13]	9	0.4	-	US		9						✓	general cognition, verbal

© 2021 Osborn AJ et al. *JAMA Network Open.*

Chuang et al., 2021[14]	47	-	-	US			47						general cognition, verbal, visuospatial, motor functioning, executive function, behaviour: externalizing/internalizing
Da Costa et al., 2006[15]	6	-	-	Australia			6				✓		general cognition
Da Costa et al., 2012[16]<br>Da Costa et al., 2013[17]	26	T1: 0.7<br>T2: 1.8	88	Australia		26	26				✓	✓	general cognition, motor functioning
Engel et al., 2012[18]	46	-	80	Germany		46							global development
Gewalli et al., 2001[19]	26	T1: 0.5<br>T2: 1.4	81	Sweden		26	26						motor functioning, verbal, visuospatial, adaptive & social skills, global development
Hashim et al., 2014[20]<br>Patel et al., 2014[21]	70	10.0	-	US			70				✓		general cognition, motor functioning, verbal, visuospatial, learning difficulties, behavior: externalizing/internalizing/total, memory: shorter term
Imahiyerobo et al., 2019[22]	77	0.4	78	US		77					✓	✓	general cognition, motor functioning, verbal
Infant Learning Project<br>Kapp-Simon et al., 2005[23]<br>Ruiz-Correa et al., 2007[24]<br>Speltz et al., 2007[25]<br>Starr et al., 2007[26]<br>Toth et al., 2008[27]<br>Naumann et al., 2012[28]<br>Starr et al., 2012[29]<br>Cradock et al., 2015[30]<br>Speltz et al., 2015[31]<br>Speltz et al., 2016[32]<br>Kapp-Simon et al., 2016[33]<br>Collett et al., 2017[34]<br>Wallace et al., 2016[35]	94	T1: 0.5<br>T2: 1.5<br>T3: 3.0<br>T4: 7.0	83	US		62	94		✓		✓	✓	general cognition, motor functioning, verbal, visuospatial, arithmetic, attention, executive function, learning difficulties, memory: shorter term, memory: longer term, behavior: externalizing/internalizing/total
Kapp-Simon et al., 1993[36]<br>Kapp-Simon et al., 1998[37]	24	T1: 0.7<br>T2: 1.5<br>T3: 4.2	86	US	4	20	20						general cognition, learning difficulties
Kljajic et al., 2019[38]<br>Kljajic et al., 2020[39]	41	11.5	68	Sweden			41					✓	general cognition, verbal, visuospatial, attention, memory: shorter term, processing speed

© 2021 Osborn AJ et al. *JAMA Network Open.*

Korpilahti et al., 2012[40]	18	3.4	90	Finland			18					✓	verbal
Lee et al., 2015[41]<br>Lee et al., 2017[42]	37	-	75	Sth Korea		37	25		✓				general cognition, motor functioning,
Magge et al., 2002[43]	16	10.3	-	US			16						learning difficulties
Mazzaferro et al., 2018[44]	5	-	-	US			5						quality of life
Moreno-Villagomez et al., 2020[45]	10	4.5	80	Mexico			10						general cognition, motor functioning, verbal, visuospatial, arithmetic, memory: shorter term, processing speed
Noetzel et al., 1985[46]	3	-	-	US				3					general cognition
Salokorpi et al., 2018[47]	40	27.4	63	Finland			40						satisfaction with appearance
Scheuerle et al., 2004[48]	3	-	-	US		3	3						verbal
Shipster et al., 2003[49]	76	0.4	80	UK				76			✓	✓	general cognition, verbal, visuospatial, learning difficulties
Speltz et al., 1993[50]	7	5.8	86	US			7				✓		behavior: externalizing/internalizing/total, self-concept
Speltz et al., 1997[51]	19	0.3	-	US		18	19						general cognition, motor functioning
Virtanen et al., 1999[52]	18	12.2	72	Finland			18						motor functioning, verbal, visuospatial, attention, memory: shorter term

Note: N = sagittal synostosis participants; descriptive statistics reflect non-syndromic metopic synostosis patient details extracted from each paper; data from non-independent papers combined & treated as a single study in analyses as appropriate. Study details above refer to the *specific* sagittal sample data extracted from each study & used in the current meta-analysis (which may differ from overall study details)

eTable 3

Studies Using Overlapping Samples - Combined and Treated as Non-independent Studies in the Current Meta-analysis

Study 'name' in meta-analysis	Papers using overlapping samples
Bellew 2005/2011/2015/2019	Bellew et al, 2005[2]
	Bellew et al, 2011[3]
	Bellew et al, 2015[4]
	Bellew et al, 2019[5]
Da Costa 2012/2013	Da Costa et al, 2012[16]
	Da Costa et al, 2013[17]
Hashim 2014/Patel 2014	Patel et al, 2014[21]
	Hashim et al, 2014[20]
Infant Learning Project (ILP)	Collett et al, 2017[34]
	Cradock et al, 2015[30]
	Kapp-Simon et al, 2005[23]
	Kapp-Simon et al, 2016[33]
	Naumann et al, 2012[28]
	Ruiz-Correa et al, 2007[24]
	Speltz et al, 2007[25]
	Speltz et al, 2015[31]
	Speltz et al, 2016[32]
	Starr et al, 2007[26]
	Starr et al, 2012[29]
	Toth et al, 2008[27]
	Wallace et al, 2016[35]
Kapp-Simon 1993/1998	Kapp-Simon et al, 1993[36]
	Kapp-Simon et al, 1998[37]
Kljajic 2019/2020	Kljajic et al, 2019[38]
	Kljajic et al, 2020[39]
Lee 2015/2017	Lee et al, 2015[41]
	Lee et al, 2017[42]

© 2021 Osborn AJ et al. *JAMA Network Open.*

eTable 4

Data Extracted From Studies Included in the Meta-analysis (Where Reported)

- Article title, year and authors
- Sample size
- Country of origin
- Age at assessment
- Sex
- Type of craniosynostosis
- Surgical status
- Type of surgical treatment
- Age at surgery
- Number of surgeries
- Criteria for surgery
- Length of post-surgery follow-up
- Sagittal synostosis diagnosis confirmation
- Status of genetic disorders
- Socio-economic status
- Family history of craniosynostosis
- Method of identification and recruitment
- Outcome
- Measures
- Outcome data

© 2021 Osborn AJ et al. *JAMA Network Open.*

eTable 5

Summary of the Cognitive Tests That Were Used by the Studies and Their Corresponding Cognitive Domain

**Domain**	**Test**
General cognition	BSID, BSID-II, BSID-III: mental development index, cognitive scale
	McCarthys Scale of Children's Abilities: general cognitive index
	K-ABC: IQ
	WPPSI-III, WPPSI-R-UK, WISC-III, WISC-IV, WASI, WAIS-R: FSIQ
	Brunet-Lezine; Nouvelle Echelle Metrique de L'Intelligence
	Cattell Infant Intelligence Scale; Stanford-Binet Intelligence Scale
Verbal Functions &	British Picture Vocabulary Scale
Language Skills	BSID, BSID-III: language , receptive, expressive
	CTOPP: phonological awareness composite
	Denver Developmental Screening Test: language
	Griffiths Mental Development Scale: hearing & speech/language
	NEPSY-II: Word generation (semantic, initial letter)
	PLS-3: receptive, expressive
	Renfrew Action Picture Test: grammar, information
	Renfrew Word Finding Vocabulary Scale
	Clinical Evaluation of Language Fundamentals: expressive, receptive
	Reynell Scales: receptive, expressive
	Token Test-II
	TOWRE: total words
	WPPSI-III, WPPSI-R-UK, WISC-III, WISC-III-UK, WISC-IV, WISC-R, WASI, WAIS-R: Verbal Comprehension Index, Verbal IQ, general language composite, information, vocabulary, similarities
	WRAT-4: reading composite, spelling
	Wechsler Fundamentals: reading-related
	Subjective/proxy-rated:
	ASQ-3: Communication; CCC-2: general communication composite
Motor functioning	Beery-VMI: total, motor co-ordination index
	BSID, BSID-II, BSID-III: psychomotor development index, fine motor, gross motor
	Griffiths Mental Development Scale: locomotor, eye/hand co-ordination
	Purdue Pegboard Test: preferred, non-preferred, both, assembly
	WPPSI-III: motor index
	Subjective/proxy-rated:
	ASQ-3: gross motor skills; fine motor skills
Visuospatial	Beery-VMI: visual perception
	Developmental Test of Visual Perception-2: motor-reduced visual perception index, visual closure
	Griffiths Mental Development Scale: performance
	Hooper Visual Organisation Test
	NEPSY-II: arrows
	Rey Complex Figure Test
	WPPSI-III, WPPSI-R-UK, WISC-III, WISC-III-UK, WISC-R, WISC-IV, WASI, WAIS-R: Performance IQ, Perceptual Reasoning Index, perceptual organisation, picture arrangement, picture completion, object assembly, block design
	Design fluency (5 point test)

© 2021 Osborn AJ et al. *JAMA Network Open.*

Attention	CCPT-3: C, detectability, omissions, commissions, perseveration, HRT, HRT-SD, Variability, HRT-BC, HRT-iC
	Corsi Block Tapping Test
	K-ABC: sequential processing index, hand movements, number recall
	Mottier Test
	TAP: alertness tonic, alertness phasic, Go/Nogo (selective attention), divided attention, sustained attention
	TEA-Ch: sky search, score!, sky search DT, score DT
	WISC-III, WISC-IV, WISC-R, WAIS-R: digit span, digits forward, letter-number sequencing, freedom from distractibility
Processing speed	WPPSI-III, WISC-III, WISC-IV, WAIS-R: processing speed index, processing speed quotient, coding, digit symbol, symbol search
Executive Function	NEPSY-II: Inhibition
	Stroop test
Arithmetic	McCarthy Scale of Children's Abilities: quantitative index
	K-ABC: arithmetic
	WRAT-4: math computation
	Wechsler Fundamentals: numerical operations
Memory – short-term	Benton's Visual Retention: Multiple Choice Form
	McCarthy Scale of Children's Abilities: memory scale
	CMS: stories (immediate, immediate thematic); word lists (learning)
	Rey Visual Design Learning Test: immediate memory span, new learning ability, memory
	Verbal learning + memory test (VLMT): immediate memory span, susceptibility to interference
	WISC-IV: working memory index
Memory – longer-term	CMS: stories (delayed, delayed recognition, delayed thematic); word lists (delayed, delayed recognition)
	CTOPP: rapid naming composite
Global development	BSID-II, BSID-III: mental/motor developmental delays, developmental delays
	Griffiths Mental Development Scale: general quotient, global development quotient
	Developmental Brunet-Lezine Scale
Learning/literacy problems	reading-related learning disability, mathematic-related learning disability, reading learning disability, spelling learning disability, literacy impairment, learning problems (<25th percentile on standardized tests)
	Subjective/proxy-rated behavioral and psychological measures
Behavior	ASEBA/CBCL externalizing index, internalizing index
	Behavior Assessment System for Children - 2: externalizing index, internalizing index
	Strengths & Difficulties Questionnaire: externalizing, internalizing
Adaptive /functional/ social	Ages & Stages Questionnaire-3: personal-social, problem solving
	Strengths & Difficulties Questionnaire: prosocial
	Griffiths Mental Development Scale: personal-social
	Behavior Assessment System for Children - 2: adaptive skills index
Satisfaction with Appearance	Visual analog scale

© 2021 Osborn AJ et al. *JAMA Network Open.*

Self-concept	Method used by Eder
Quality of Life	TACQOL: EMOPOS, EMONEG, body, motor, auto, cognition, social
	WHOQOL: physical health, psychological, social, environmental
Attention	ASEBA/CBCL: parent/teacher
Executive function	BRIEF: behavioural regulation index, metacognition index, global executive composite
Memory - shorter-term	BRIEF: working memory

Note: all cognitive measures are categorised in meta-analyses as ‘objective’ except where noted above; general cognition = test scores on measures of IQ; global developmental delay = poor developmental progress in multiple domains (e.g cognition, language, motor skills); BSID = Bayley Scales of Infant Development; K-ABC = Kaufman Assessment Battery for Children; WASI: Wechsler Abbreviated Scale Intelligence; WPPSI: Wechsler Preschool and Primary Scale of Intelligence; WISC = Wechsler Intelligence Scale for Children; WAIS = Wechsler Adult Intelligence Scale; CTOPP = Comprehensive Test of Phonological Processing; WRAT-4 = Wide Range Achievement Test 4; TOWRE = Test of Word Reading Efficiency; PLS = Preschool Language Scale; NEPSY-II = A Developmental Neuropsychological Assessment; CCC-2 = Children’s Communication Checklist-2; Tea-CH = Test of Everyday Attention in Children; BRIEF = Behavioral Rating Inventory of Executive Function; CMS = Children’s Memory Scale; Beery-VMI = Beery-Buktenica Test of Visual-Motor Integration; ASQ = Ages & Stages Questionnaire ; CCPT = Connor’s Continuous Performance Test; TAP = Tests for Attentional Performance; ASEBA = Achenbach System of Empirically Based Assessment; CBCL = Child Behavior Checklist; TACQOL = TNO AZL Children’s Quality of Life; WHOQOL = World Health Organization Quality of Life.

© 2021 Osborn AJ et al. *JAMA Network Open.*

eTable 6

Adapted NIH Quality Assessment of Observational Cohort and Cross-Sectional Studies

Study (lead author, year)	Item 1	Item 2	Item 3	Item 4a	Item 4b	Item 5	Item 6	Item 7	Item 8	Item 9	Item 10	Item 11
Arnaud 1995 [1]	Y	N	Y	Y	Y	N	Y	N	N	NR	NA	N
Bellew $_{2005, 2011, 2015, 2019}$ [2-5]	Y	Y	NR	Y	N	N	Y	Y	Y	NR	N	N
Byun 2018 [7]	N	N	NR	Y	Y	N	Y	N	Y	NR	NA	N
Care 2019 [9]	N	Y	Y	Y	Y	N	Y	Y	Y	NR	NA	N
Chandler 2020 [10]	Y	N	NR	NR	Y	N	Y	Y	Y	NR	NA	Y
Chieffo 2020 [12]	Y	N	Y	NR	N	N	Y	Y	N	NR	N	N
Chuang 2021 [13]	Y	Y	N	Y	Y	N	Y	Y	Y	NR	NA	Y
Da Costa 2006 [15]	Y	N	NR	NR	Y	N	Y	Y	Y	NR	NA	Y
Da Costa $_{2012/2013}$ [16,17]	Y	N	Y	NR	Y	N	Y	Y	Y	NR	NA	N
Engel 2012 [18]	Y	Y	NR	Y	Y	N	Y	Y	Y	NR	NA	N
Gewalli 2001 [19]	Y	Y	NR	Y	N	N	Y	Y	Y	NR	Y	N
Hashim 2014 / Patel 2014 [20,21]	Y	Y	Y	Y	Y	N	Y	Y	Y	NR	NA	Y
Imahiyerobo 2019 [22]	Y	Y	Y	Y	N	N	Y	N	Y	NR	NA	Y
Kapp-Simon $_{1993/1998}$ [36,37]	Y	N	Y	Y	Y	N	Y	Y	Y	NR	N	N
Kljajic $_{2019/2020}$ [38,39]	Y	Y	Y	Y	Y	N	Y	Y	Y	NR	NA	Y
Korpilahti 2012 [40]	Y	N	NR	NR	Y	N	Y	Y	Y	NR	NA	N
Lee $_{2015 / 2017}$ [41,42]	Y	N	NR	Y	N	N	Y	Y	Y	NR	N	N
Magge 2002 [43]	Y	Y	N	Y	Y	N	Y	Y	Y	NR	NA	N
Mazzaferro 2018 [44]	Y	N	N	NR	Y	N	Y	Y	Y	NR	NA	Y
Moreno-Villagomez 2020 [45]	Y	N	NR	NR	Y	N	Y	Y	Y	NR	NA	N
Noetzel 1985 [46]	N	N	NR	Y	N	N	Y	N	N	NR	NA	N
Scheuerle 2004 [48]	N	N	NR	N	N	N	Y	Y	N	NR	NA	N
Shipster 2003 [49]	Y	Y	Y	Y	Y	N	Y	Y	Y	NR	NA	N

NIH = National Institutes of Health; Y = yes; N = no; NR = not reported; NA = not applicable

1. Was the research question or objective in this paper clearly stated?
2. Was the study population clearly specified & defined (including demographics, location and time period)?
3. Was the participation rate of eligible persons at least 50%?
4. Were all the subjects selected or recruited from the same or similar populations (including the same time period)?
5. Were inclusion and exclusion criteria for being in the study prespecified, reported and applied uniformly to all participants?
6. Was a sample size justification, power description, or variance and effect estimates provided?
7. Was sagittal synostosis diagnosed prior to the outcome(s) being measured?

© 2021 Osborn AJ et al. *JAMA Network Open.*

8. Was the diagnosis of sagittal synostosis (independent variable) clearly defined, valid, reliable, and implemented consistently across all study participants?
9. Were the outcome measures (dependent variables) clearly defined, valid, reliable, and implemented consistently across all study participants?
10. Were the outcome assessors blinded to the exposure status of participants?
11. Was loss to follow-up after baseline 20% or less?
12. Were key potential confounding variables measured and adjusted statistically for their impact on the relationship between exposure(s) and outcome(s)?

© 2021 Osborn AJ et al. *JAMA Network Open.*

eTable 7

NIH Quality Assessment of Case-Control Studies

Study (lead author, year)	Item 1	Item 2	Item 3	Item 4	Item 5	Item 6	Item 7	Item 8	Item 9	Item 10	Item 11	Item 12a	Item 12b	Item 12c
Boltshauser, 2003 [6]	Y	N	N	NR	NR	Y	NR	NR	Y	Y	NR	N	N	NA
Cabrejo, 2019 [8]	Y	N	N	NR	Y	Y	NR	NR	Y	Y	NR	N	Y	N
Chieffo, 2010 [11]	Y	N	N	NR	NR	Y	NR	NR	Y	Y	NR	N	Y	N
Chuang, 2018 [13]	N	N	N	NR	NR	Y	NR	NR	Y	N	NR	N	N	NA
Infant Learning Project [23-37]	Y	Y	N	Y	Y	Y	NR	NR	Y	Y	NR	Y	Y	Y
Salokorpi, 2018 [47]	Y	Y	N	Y	Y	Y	NR	NR	Y	N	NR	N	Y	N
Speltz, 1993 [50]	Y	N	N	NR	NR	Y	NR	NR	Y	Y	NR	Y	Y	NR
Speltz, 1997 [51]	Y	N	N	NR	Y	Y	NR	NR	Y	Y	NR	Y	Y	Y
Virtanen, 1999 [52]	Y	Y	N	Y	Y	Y	NR	NR	Y	Y	NR	N	Y	N

NIH = National Institutes of Health; Y = yes; N = no; NR = not reported; NA = not applicable

1. Was the research question or objective in this paper clearly stated & appropriate?
2. Was the study population clearly specified & defined (including details of demographics, time period, location)?
3. Did the authors include a sample size justification?
4. Were controls selected or recruited from the same or similar population that gave rise to the cases (including the same timeframe)?
5. Were the definitions, inclusion and exclusion criteria, algorithms or processes used to identify or select cases and controls valid, reliable, and implemented consistently across all study participants?
6. Were the cases clearly defined and differentiated from controls?
7. If less than 100 percent of eligible cases and/or controls were selected for the study, were the cases and/or controls randomly selected from those eligible?
8. Was there use of concurrent controls?
9. Was the diagnosis of sagittal synostosis clearly defined, valid, reliable, and implemented consistently (including the same time period) across all study participants?
10. Were the outcome measures (dependent variables) clearly defined, valid, reliable, and implemented consistently across participants?
11. Were the assessors of outcomes blinded to the case or control status of participants?
12. Were key potential confounding variables measured and adjusted statistically in the analyses?
13. Were cases and controls matched?
14. If matching was used, did the investigators account for matching during study analysis?

© 2021 Osborn AJ et al. *JAMA Network Open.*

eTable 8

Prediction Intervals for Pooled Analyses With ≥5 Included Studies

Post-surgical domain	No. of studies	Mean g_w	CI (95%) lower limit	CI (95%) upper limit	PI (95%) lower limit	PI (95%) upper limit
General cognition: norms	9	0.01	-0.26	0.27	-0.86	0.87
Motor functioning: norms	7	0.01	-0.31	0.34	-1.09	1.12
Verbal abilities: norms	7	0.23	-.01	0.47	-0.51	0.97
Visuospatial: norms	6	0.31	0.18	0.44	0.12	0.49

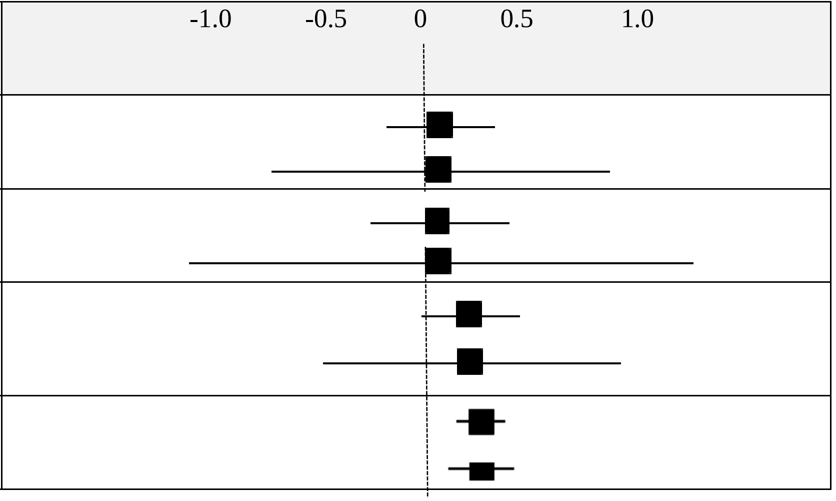

Note: CI = confidence intervals; PI = prediction intervals

© 2021 Osborn AJ et al. *JAMA Network Open.*

eTable 9

Outcomes of Mixed (Conservatively Managed + Presurgical +Postsurgical) Samples of Children With Sagittal Synostosis

COMBINED SAMPLES (combined conservatively managed, pre- and post-surgery participants)

	Standardized Mean Group Differences										Prevalence rates					
Domain	*Comparison data*	$N_{studies}$	N_{SS}	*Hedges' g*	*Lower 95% CI*	*Upper 95% CI*	*p*	I^2	*tau*	N_{fs}	$N_{studies}$	N_{SS}	Prevalence (95% CI)	$N_{studies}$	N_{SS}	Odds ratio (95% CI) logarithmic scale
General cognition	norms	3	185	-.33	-1.17	.51	.44	95.08	.72	2	2	73	0.20 (0.12 – 0.30)			
Motor functioning	norms	1	67	-.93	-1.18	-.69	**.00**	-	-	-						
Verbal abilities	norms	2	123	.12	-.24	.49	.51	47.63	.19	0	1	76	0.37 (0.27 – 0.48)			
Visuospatial abilities	norms	2	84	-.03	-.50	.44	.90	65.53	.28	0						

© 2021 Osborn AJ et al. *JAMA Network Open.*

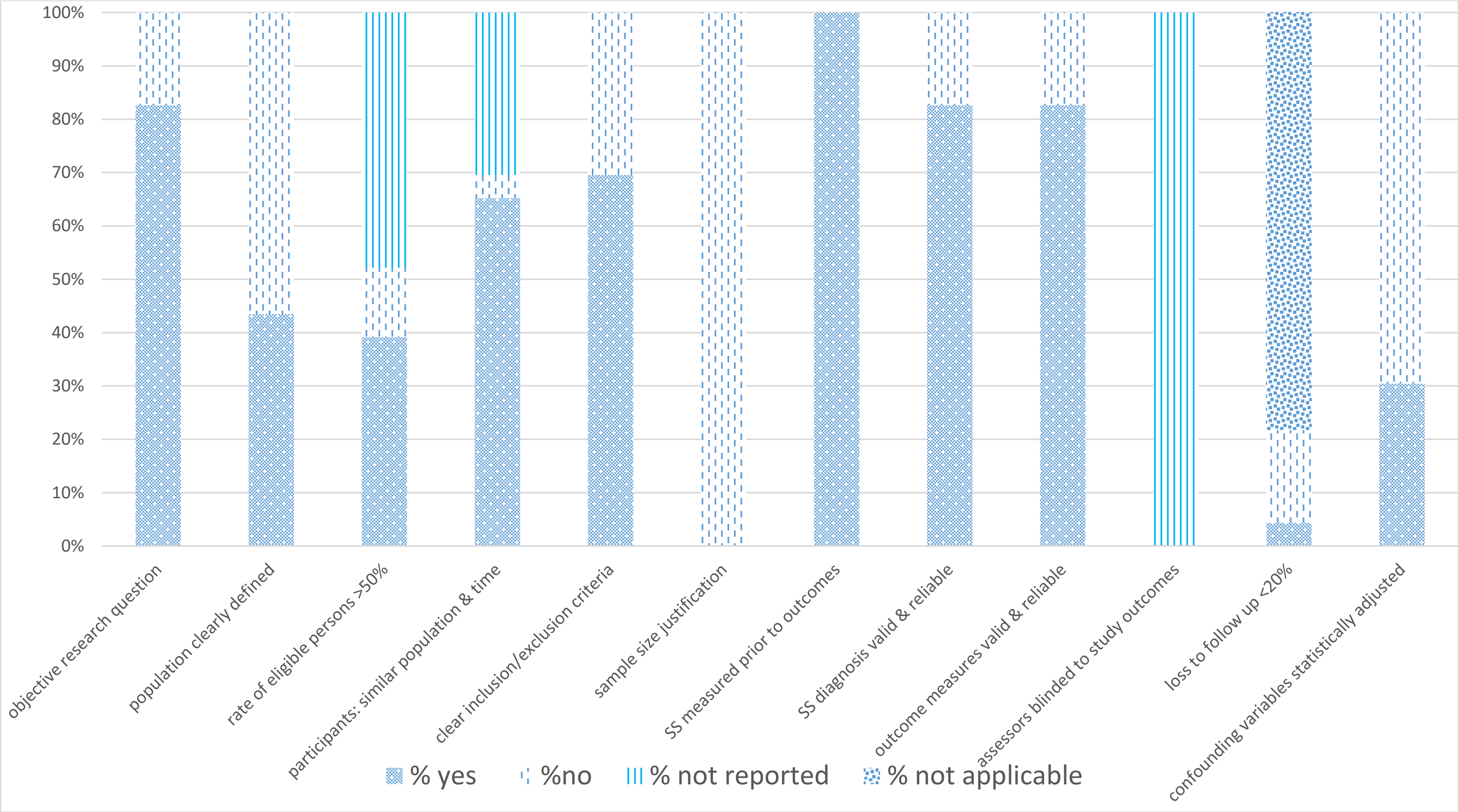

eFigure 1

Percentage of Observational Cross-Sectional and Cohort Studies Meeting Each of the Adapted NIH Study Quality Criteria ($N_{studies}$ = 23).
SS = sagittal synostosis

© 2021 Osborn AJ et al. *JAMA Network Open.*

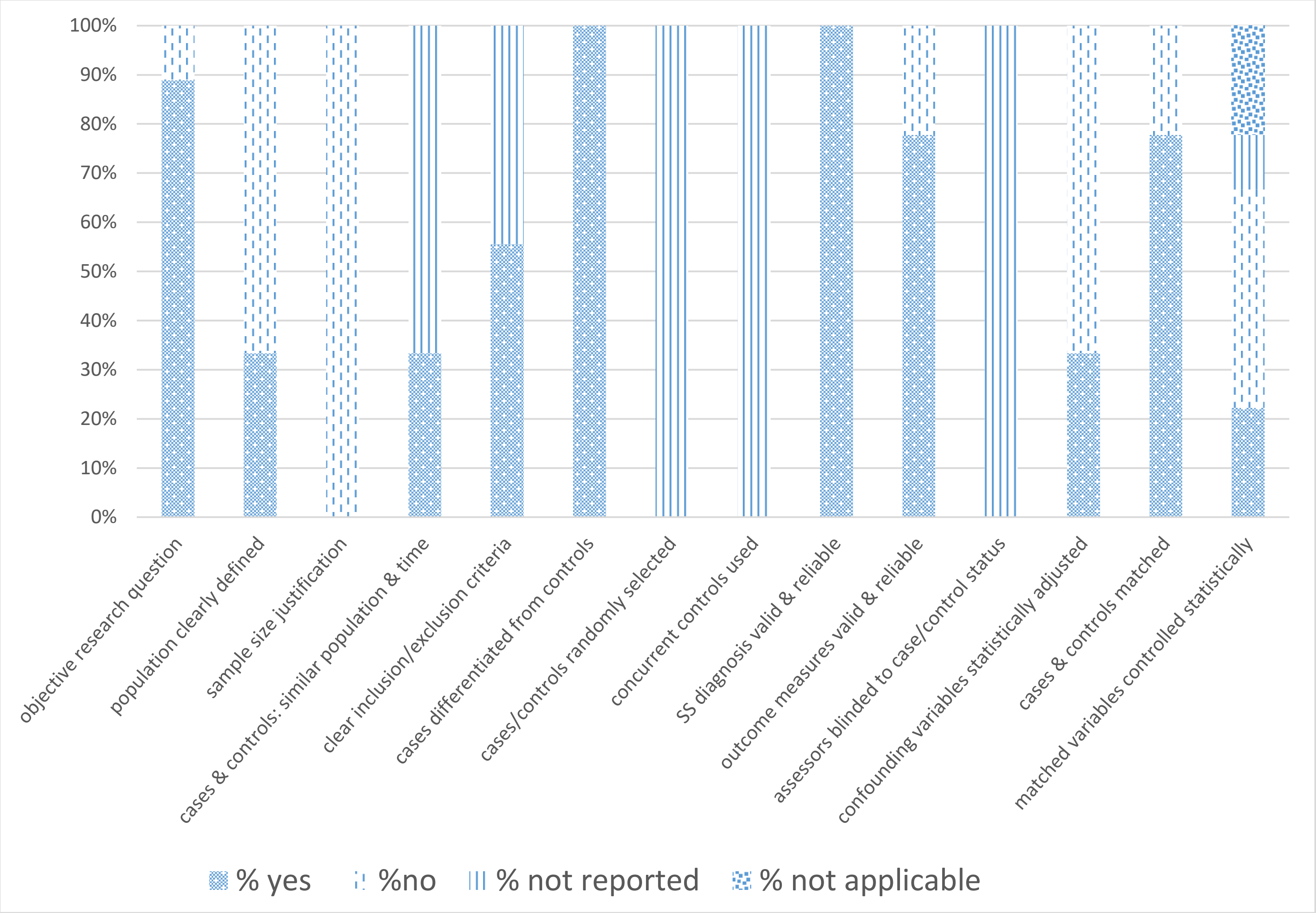

eFigure 2

Percentage of Case-Control Studies Meeting Each of the Adapted NIH Study Quality Criteria ($N_{studies}$ = 9). SS = sagittal synostosis.

© 2021 Osborn AJ et al. *JAMA Network Open.*

CONSERVATIVELY MANAGED SAGITTAL SYNOSTOSIS

Study details		Standardized Mean Group Differences					Prevalence Rates	
Authors	*N*	*Comparisons with healthy peers*		*Comparisons with norms*		*Hedges' g and 95% CI*	*Prevalence and 95% CI (single group studies)*	*Odds ratio and 95% CI (2 group studies; logarithmic scale)*
Objective measures		*g*	*p*	*g*	*p*	-2.0 -1.0 0.0 1.0 2.0<br>**SS outcomes worse** / **SS outcomes better**	0 25 50 75 100<br>**→ increasing prevalence**	0.01 0.1 1 10 100<br>**SS outcomes better** / **SS outcomes worse**
**General cognition**								
Arnaud, [1] 1995	51						4%	
Boltshauser et al, [6] 2003	30	-.08	.79				7%	
Bellew et al, (2005-2019)*	13			.13	.64			
Kapp-Simon 1993/1998*	4			.40	.42			
OVERALL	**98**			**.19**	**.43**		**5%**	
**Motor functioning**								
Bellew et al, (2005-2019)*	13			-.15	.59			
**Verbal abilities/ language**								
Bellew et al, (2005-2019)*	14			.09	.74			
Boltshauser et al, [6] 2003	14						7%	0.33
**Visuospatial abilities**								
Bellew et al, (2005-2019)*	14			.02	.93			
Boltshauser et al, [6] 2003							7%	0.69
**Adaptive & social skills**								
Bellew et al, (2005-2019)*	13			-.14	.62			
**Arithmetic**								
Boltshauser et al, [6] 2003	18							0.71
**Attention**								
Boltshauser et al, [6] 2003	19						13%	1.04
**Executive function**								
Boltshauser et al, [6] 2003	12						8%	
**Global development**								
Bellew et al, (2005-2019)*	23			.58	.01			
**Memory: shorter term**								
Boltshauser et al, [6] 2003	18						9%	0.70
**Processing speed**								
Boltshauser et al, [6] 2003	14						7%	0.31

© 2021 Osborn AJ et al. *JAMA Network Open.*

Subjective measures				
**Behavior- externalizing**				
Boltshauser et al, [6] 2003	24	-.25	.22	
**Behavior: internalizing**				
Boltshauser et al, [6] 2003	24	-.30	.14	
**Behavior: overall**				
Boltshauser et al, [6] 2003	24	-.29	.16	
**Quality of life**				
Boltshauser et al, [6] 2003	24	.00	1.0	

Note: *N* refers to sagittal synostosis participants; * = details for studies comprising the same participants across multiple papers are provided in eTable 2 in the Supplement; green forest plots = comparisons between SS and healthy norms; black forest plots = comparisons between SS and normative data.

eFigure 3

Outcomes of Children With Conservatively Managed Sagittal Synostosis.

PRE-SURGICAL SAGITTAL SYNOSTOSIS

Study details		Standardized Mean Group Differences				Prevalence Rates
Authors	*N*	*Comparisons with healthy peers*		*Comparisons with norms*		*Prevalence and 95% CI*
Objective measures		*g*	*p*	*g*	*p*	
**General cognition**						
Chuang et al, [13] 2018	9	.31	.45			
Da Costa et al, 2012/2013*	26			-.24	.22	
ILP (2005 – 2017)*	62	-.46	.00			
Imahiyerobo et al, [22] 2019	77			.52	.00	
Kapp-Simon 1993/1998*	20			.17	.46	
Lee et al, 2015/2017*	37			-1.03	.00	37%
Speltz et al, [51] 1997	18	.29	.37			
OVERALL	**249**	**-.28**	**.15**	**-.15**	**.70**	
**Motor functioning**						
Bellew et al, (2005-2019)*	28	-.44	.10			
Chieffo et al, [12] 2020	87					11%
Da Costa et al, 2012/2013*	26			-.82	.00	
Gewalli et al, [19] 2001	26			.44	.03	
ILP (2005 – 2017)*	62	-.41	.01			
Imahiyerobo et al, [22] 2019	77			.21	.07	
Lee et al, 2015/2017*	37			-1.04	.00	26%
Speltz et al, [51] 1997	18	-.47	.15			
OVERALL	**361**	**-.42**	**.00**	**-.30**	**.41**	**17%**
**Verbal abilities / language**						
Bellew et al, (2005-2019)*	28	-.01	.96			
Chieffo et al, [12] 2020	87					8%
Chuang et al, [13] 2018	9	.32	.45			
Gewalli et al, [19] 2001	26			.24	.23	
ILP (2005 – 2017)*	62	-.03	.85			
Imahiyerobo et al, [22] 2019	77			.15	.22	
Scheuerle 2004	3					33%
OVERALL	**292**	**.01**	**.96**	**.17**	**.09**	**13%**
**Visuospatial abilities**						
Bellew et al, (2005-2019)*	28	-.09	.73			
Chieffo et al, [12] 2020	87					7%
Gewalli et al, [19] 2001	26			.44	.03	
OVERALL	**141**					
**Adaptive & social skills**						
Bellew et al, (2005-2019)*	28	-.21	.44			
Chieffo et al, [12] 2020	87					6%
Gewalli et al, [19] 2001	26			.21	.30	
OVERALL	**141**					
**Global development**						
Arnaud, [1] 1995	142					8%
Bellew et al, (2005-2019)*	28	-.31	.25			
Chieffo et al, [12] 2020	87					25%
Engel et al, [18] 2012	46					17%
Gewalli et al, [19] 2001	26			.30	.13	8%
Imahiyerobo et al, [22] 2019	77					18%

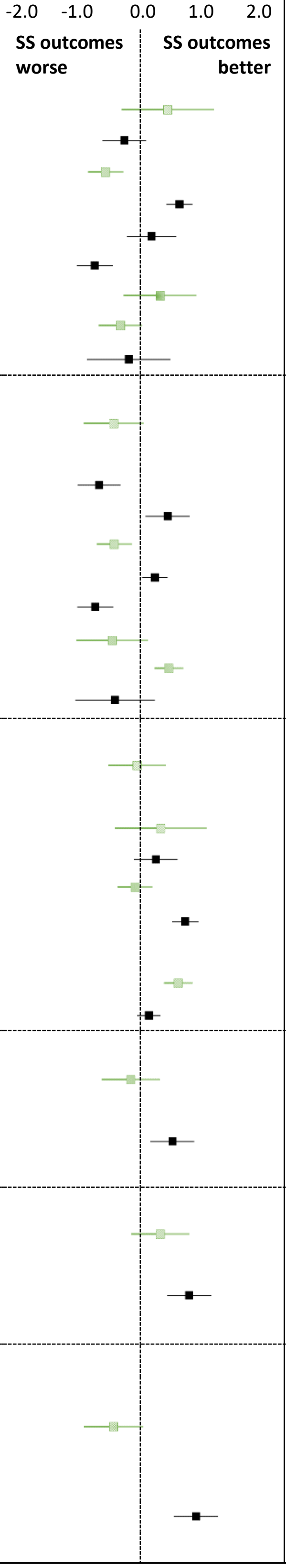

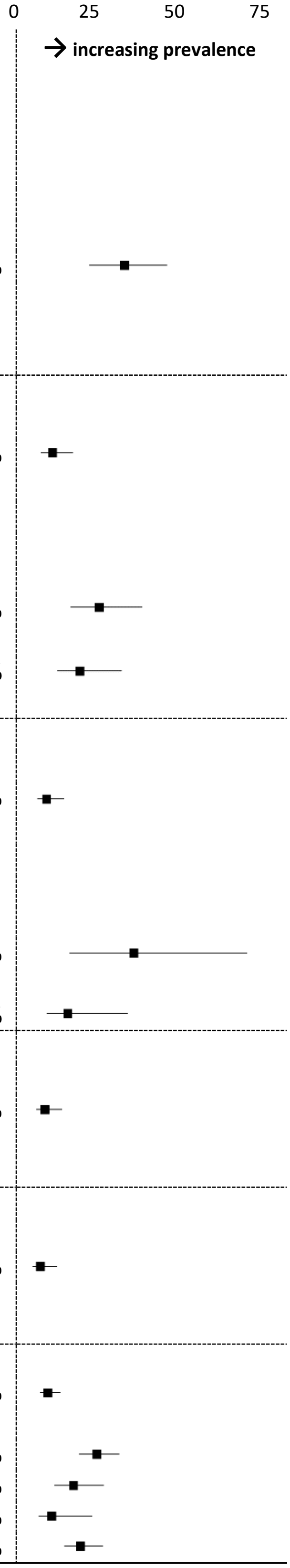

© 2021 Osborn AJ et al. *JAMA Network Open.*

Note: *N* refers to sagittal synostosis participants; ILP = Infant Learning Project combined papers; * = details for studies comprising the same participants across multiple papers are provided in eTable 2 in the Supplement; green forest plots = comparisons between SS and healthy norms; black forest plots = comparisons between SS and normative data.

eFigure 4

Presurgical Functioning of Children With Sagittal Synostosis: Had Not Yet Undergone Surgery Prior to Being Assessed.

© 2021 Osborn AJ et al. *JAMA Network Open.*

POST-SURGICAL SAGITTAL SYNOSTOSIS

Study details		Standardized Mean Group Differences				Prevalence Rates	
Authors	*N*	*Comparisons with healthy peers* *g*	*p*	*Comparisons with norms* *g*	*p*	*Prevalence and 95% CI (single group studies)*	*Odds ratio and 95% CI (2 group studies; logarithmic scale)*
Objective measures							
**General cognition**							
Arnaud, [1] 1995	47					11%	
Bellew et al, (2005-2019)*	54			.10	.59	28%	
Chuang et al, [14] 2021	47			.43	.00		
Da Costa et al, [15] 2006	6			-.03	.94		
Da Costa et al, 2012/2013*	26			-.42	.10		
Hashim/Patel et al, 2014*	70			.52	.00		
ILP (2005 – 2017)*	94	-.28	.04				
Kapp-Simon et al, 1993/1998*	20			-.13	.63		
Kljajic et al, 2019/2020*	41			-.25	.11		
Lee et al, 2015/2017*	25			-.38	.06	22%	
Moreno-Villagomez et al, [45] 2020	10			.01	.98		
Speltz et al, [51] 1997	19	.21	.51				
OVERALL	**459**	**-.12**	**.60**	**.01**	**.96**	**20%**	
**Motor functioning**							
Bellew et al, (2005-2019)*	50			.58	.00		
Chieffo et al, [12] 2020	63					6%	
Chuang et al, [14] 2021	47			.27	.07		
Da Costa et al, 2012/2013*	16			-.89	.00		
Gewalli et al, [19] 2001	26			.16	.42		
Hashim/Patel et al, 2014*	70			.27	.03		
ILP (2005 – 2017)*	94	-.22	.12				
Lee 2015/2017	25			-.41	.04	22%	
Moreno-Villagomez et al, [45] 2020	10			-.14	.66		
Speltz et al, [51] 1997	19	.00	1.0				
Virtanen et al, [52] 1999	18	-.15	.65				
OVERALL	**438**	**-.18**	**.13**	**.01**	**.93**	**12%**	
**Verbal abilities / language**							

Hedges' g and 95% CI axis: -2.0, -1.0, 0.0, 1.0, 2.0; SS outcomes worse / SS outcomes better. Prevalence axis: 0, 25, 50, 75; → increasing prevalence. Odds ratio axis: 0.01, 0.1, 1, 10, 100.

© 2021 Osborn AJ et al. *JAMA Network Open.*

Bellew et al, (2005-2019)*	54			.51	.00		
Chuang et al, [14] 2021	47			.57	.00		
Cabrejo et al, [8] 2019	10	-1.20	.01				
Chieffo et al, [12] 2020	63					17%	
Gewalli et al, [19] 2001	26			.02	.92		
Hashim/Patel et al, 2014*	70			.46	.00		
ILP (2005 – 2017)*	94	-.15	.27				
Kljajic et al, 2019/2020*	41			-.16	.31		
Korpilahti et al, [40] 2012	18			.09	.69	11%	
Moreno-Villagomez et al, [45] 2020	10			-.15	.65		
Scheuerle et al, [48] 2004	3					13%	
Virtanen et al, [52] 1999	18	-.90	.01				
OVERALL	**454**	**-.65**	**.06**	**.23**	**.06**	**15%**	
**Visuospatial abilities**							
Bellew et al, (2005-2019)*	54			.13	.43		
Cabrejo et al, [8] 2019	10	-.32	.46				
Chieffo et al, [11] 2010	35						4.55
Chieffo et al, [12] 2020	63					5%	
Chuang et al, [14] 2021	47			.29	.05		
Gewalli et al, [19] 2001	26			.35	.08		
Hashim/Patel et al, 2014*	70			.49	.00		
ILP (2005 – 2017)*	74	.02	.90				
Kljajic et al, 2019/2020*	41			.26	.10		
Moreno-Villagomez et al, [45] 2020	10			-.03	.92		
Virtanen et al, [52] 1999	18	-.43	.19				
OVERALL	**448**	**-.07**	**.56**	**.31**	**.00**		
**Adaptive & social skills**							
Bellew et al, (2005-2019)*	50			.22	.13		
Chieffo et al, [12] 2020	63					3%	
Gewalli et al, [19] 2001	26			-.13	.53		
OVERALL	**139**			**.07**	**.67**		
**Arithmetic**							
ILP (2005 – 2017)*	75	-.21	.12				
Moreno-Villagomez et al, [45] 2020	10			-.24	.45		
OVERALL	**85**						
**Attention**							

© 2021 Osborn AJ et al. *JAMA Network Open.*

ILP (2005 – 2017)*	76	-.11	.43				
Kljajic et al, 2019/2020*	38			-.51	.00		
Virtanen et al, [52] 1999	18	-.82	.02				
OVERALL	**132**	**-.40**	**.26**				
**Executive function**							
ILP (2005 – 2017)*	75	-.18	.19				
**Global development**							
Bellew et al, (2005-2019)*	50			.53	.00		
Gewalli et al, [19] 2001	26			.09	.64	15%	
OVERALL	**76**			**.34**	**.13**		
**Learning difficulties**							
Chuang et al, [14] 2021	47					12%	
ILP (2005 – 2017)*	70						0.64
Hashim/Patel et al, 2014*	70					23%	
Kapp-Simon et al, 1993/1998*	10					13%	
Magge et al, [43] 2002	16					50%	
OVERALL	**213**					**22%**	
**Memory: shorter term**							
ILP (2005 – 2017)*	74	-.24	.09				
Kljajic et al, 2019/2020*	41			-.49	.00		
Moreno-Villagomez et al, [45] 2020	10			-.28	.38		
Virtanen et al, [52] 1999	18	.00	1.0				
OVERALL	**143**	**-0.20**	**.12**				
				**-.45**	**.00**		
**Memory: longer term**							
ILP (2005 – 2017)*	74	-.18	.20				
**Processing speed**							
Kljajic et al, 2019/2020*	41			-.65	.00		
Moreno-Villagomez et al, [45] 2020	7			.27	.48		
OVERALL	**48**			**-.26**	**.57**		
Subjective measures							
**Motor functioning**							
Care et al, [53] 2019	138					10%	
**Verbal abilities / language**							
ILP (2005 – 2017)*	70	-.01	.93				
Care et al, [53] 2019	138					9%	
OVERALL	**208**						

© 2021 Osborn AJ et al. *JAMA Network Open.*

**Adaptive & social skills**							
Care et al, [53] 2019	140					12%	
Chandler et al, [10] 2020	75			.15	.19		
OVERALL	**215**						
**Attention**							
ILP (2005 – 2017)*	76	-.02	.87				
**Behavior- externalizing**							
Care et al, [53] 2019	94					26%	
Chandler et al, [10] 2020	75			.00	.97		
Chuang et al, [14] 2021	47			-.22	.13		
Hashim/Patel et al, 2014*	70			-.22	.07		
ILP (2005 – 2017)*	76	-.01	.93				1.48
Speltz et al, [50] 1993	7	-.39	.42				
OVERALL	**369**	**-.04**	**.76**	**-.14**	**.08**		
**Behavior: internalizing**							
Care et al, [53] 2019	94					14%	
Chandler et al, [10] 2020	75			-.11	.37		
Chuang et al, [14] 2021	47			-.04	.77		
Hashim/Patel et al, 2014*	70			-.06	.64		
ILP (2005 – 2017)*	76	-.08	.54				1.17
Speltz et al, [50] 1993	7	-.66	.17				
OVERALL	**369**	**-.19**	**.40**	**-.07**	**.32**		
**Behavior: total**							
Care et al, [53] 2019	94					22%	
Chandler et al, [10] 2020	75			.17	.30		
ILP (2005 – 2017)*	76	-.05	.72				1.52
Speltz et al, [50] 1993	7	.49	.31				
OVERALL	**252**	**.02**	**.91**				
**Executive function**							
ILP (2005 – 2017)*	75	.04	.79				
Chandler et al, [10] 2020	75			-.18	.14		
Chuang et al, [14] 2021	47			-.44	.00		
Hashim/Patel et al, 2014*	70			-.60	.00		
OVERALL	**267**			**-.40**	**.00**		
**Memory: short-term**							
Chandler et al, [10] 2020	75			-.19	.11		

© 2021 Osborn AJ et al. *JAMA Network Open.*

Chuang et al, [14] 2021	47			-.41	.01
Hashim/Patel et al, 2014*	70			-.58	.00
OVERALL	**192**			**-.39**	**.00**
**Quality of life**					
Mazzaferro et al, [44] 2018	5			1.29	.00
**Satisfaction with appearance**					
Salokorpi et al, [54] 2018	40	-.06	.78		
**Self-concept**					
Speltz et al, [50] 1993	7	-.03	.94		

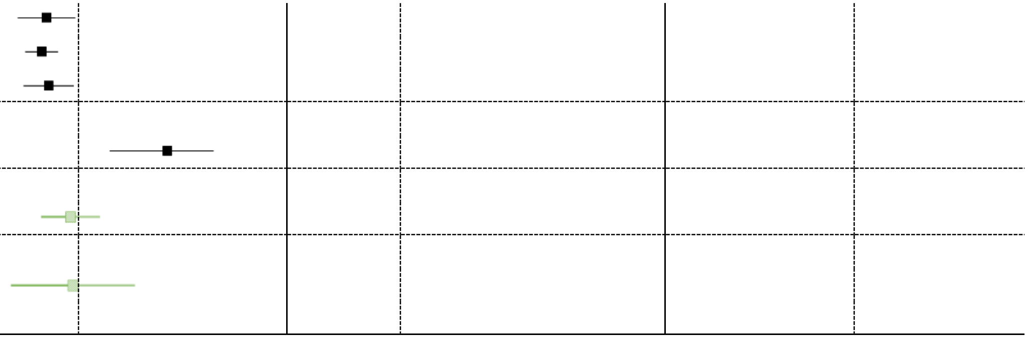

Note: *N* refers to sagittal synostosis participants; ILP = Infant Learning Project combined papers; * = details for studies comprising the same participants across multiple papers are provided in eTable 2 in the Supplement; green forest plots = comparisons between SS and healthy norms; black forest plots = comparisons between SS and normative data.

eFigure 5

Outcomes of Children With Operated Sagittal Synostosis: Had Undergone Surgery Prior to Being Assessed.

© 2021 Osborn AJ et al. *JAMA Network Open.*

eFigure 6

Pooled Analyses for Each Cognitive, Behavioral and Psychological Domain, Partitioned According to Surgical Status (Conservatively Managed, Presurgical, Postsurgical) and Age at Assessment: A (0 – 0.11); B (1:0 – 2:11); C (3:0 – 6:11); D (7:0 – 17:11); E (>18:0)

OBJECTIVE MEASURES

3a. General cognition: conservatively managed samples

Group by Age category	Study name	Surgical status	Comparison type	Measure	Age at assessment	Hedges's g	Standard error	Variance	Lower limit	Upper limit	Z-Value	p-Value
						Statistics for each study						
A (0 - 0:11)	Kapp-Simon 1993/1998 A	CM	norms	BSID:MDI	8.1 mths	0.400	0.501	0.251	-0.581	1.381	0.799	0.424
A (0 - 0:11)						0.400	0.501	0.251	-0.581	1.381	0.799	0.424
D (7:0 - 17:11)	Bellew 2005/2011/2015/2019	CM	norms	WISC-IV: FSIQ	10 yrs	0.127	0.278	0.077	-0.418	0.672	0.455	0.649
D (7:0 - 17:11)	Boltshauser 2003	CM	peers	Combined	9.25 yrs	-0.081	0.299	0.089	-0.666	0.505	-0.270	0.787
D (7:0 - 17:11)						0.030	0.204	0.041	-0.369	0.429	0.149	0.881

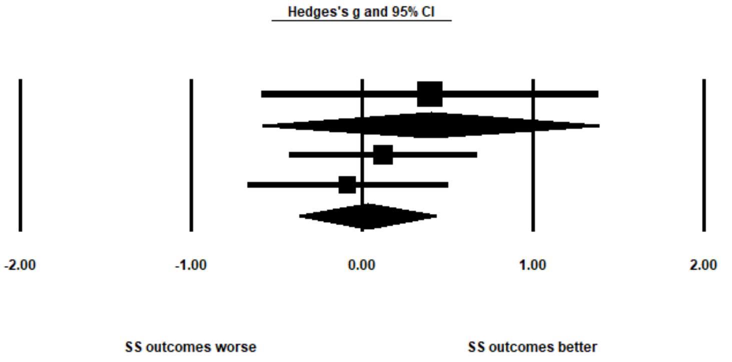

3b. General cognition: pre-surgical samples

Group by Age category	Study name	Surgical status	Comparison type	Measure	Age at assessment	Hedges's g	Standard error	Variance	Lower limit	Upper limit	Z-Value	p-Value
						Statistics for each study						
A (0 - 0:11)	Chuang 2018	pre-surgery	peers	BSID: cognitive	133 days	0.314	0.420	0.176	-0.508	1.137	0.749	0.454
A (0 - 0:11)	Da Costa 2012/2013	pre-surgery	norms	BSID-II: MDI	8.2 mths	-0.241	0.198	0.039	-0.628	0.146	-1.221	0.222
A (0 - 0:11)	ILP	pre-surgery	peers	BSID-II: MDI	7 mths	-0.463	0.157	0.025	-0.769	-0.156	-2.955	0.003
A (0 - 0:11)	Imahiyerobo 2019	pre-surgery	norms	BSID-III: cognitive	5.1 mths	0.517	0.117	0.014	0.288	0.745	4.423	0.000
A (0 - 0:11)	Kapp-Simon 1993/1998 A	pre-surgery	norms	BSID: MDI	8.1 mths	0.167	0.225	0.051	-0.275	0.608	0.740	0.459
A (0 - 0:11)	Speltz 1997	pre-surgery	peers	BSID: MDI	4.1 mths	-0.292	0.324	0.105	-0.926	0.342	-0.902	0.367
A (0 - 0:11)						-0.005	0.207	0.043	-0.410	0.401	-0.023	0.982
B (12:0 - 2:11)	Lee 2015/2017	pre-surgery	norms	BSID-II: MDI	17.5 mths	-1.033	0.167	0.028	-1.361	-0.706	-6.187	0.000
B (12:0 - 2:11)						-1.033	0.167	0.028	-1.361	-0.706	-6.187	0.000

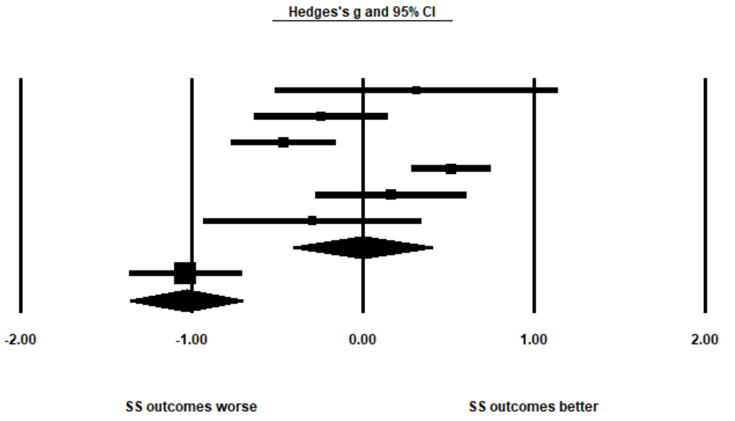

© 2021 Osborn AJ et al. *JAMA Network Open.*

3c. General cognition: post-surgical samples

Group by Age category	Study name	Surgical status	Comparison type	Measure	Age at assessment	Hedges's g	Standard error	Variance	Lower limit	Upper limit	Z-Value	p-Value
B (12:0 - 2:11)	Da Costa 2012/2013	post-surgery	norms	BSID-II: MDI	21.2 mths	-0.419	0.251	0.063	-0.911	0.073	-1.668	0.095
B (12:0 - 2:11)	ILP B	post-surgery	peers	BSID-II: MDI	18 mths	-0.180	0.132	0.017	-0.438	0.078	-1.366	0.172
B (12:0 - 2:11)	Kapp-Simon 1993/1998 B	post-surgery	norms	BSID: MDI	21 mths	-0.167	0.237	0.056	-0.632	0.298	-0.704	0.481
B (12:0 - 2:11)	Lee 2015/2017	post-surgery	norms	BSID-II: MDI	31.5 mth	-0.383	0.201	0.041	-0.778	0.012	-1.900	0.057
B (12:0 - 2:11)	Speltz 1997 A	post-surgery	peers	BSID: MDI	12.4 mths	0.337	0.320	0.102	-0.290	0.965	1.054	0.292
B (12:0 - 2:11)	Speltz 1997 B	post-surgery	peers	BSID: MDI	24.4 mths	0.085	0.322	0.104	-0.546	0.716	0.263	0.793
B (12:0 - 2:11)						-0.186	0.089	0.008	-0.360	-0.012	-2.092	0.036
C (3:0 - 6:11)	ILP C	post-surgery	peers	BSID-II: MDI	3 yrs	-0.356	0.125	0.016	-0.601	-0.111	-2.851	0.004
C (3:0 - 6:11)	Kapp-Simon 1993/1998 C	post-surgery	norms	BSID: MDI	50 mths	-0.089	0.290	0.084	-0.657	0.480	-0.305	0.760
C (3:0 - 6:11)	Moreno-Villagomez 2020	post-surgery	norms	WPPSI-III: FSIQ	54.2 mths	0.007	0.317	0.100	-0.615	0.628	0.021	0.983
C (3:0 - 6:11)						-0.277	0.108	0.012	-0.489	-0.066	-2.568	0.010
D (7:0 - 17:11)	Bellew 2005/2011/2015/2019	post-surgery	norms	WISC-IV: FSIQ	Combined	0.095	0.177	0.031	-0.253	0.442	0.534	0.593
D (7:0 - 17:11)	Da Costa 2006	post-surgery	norms	WISC-III: FSIQ	10.9 yrs	-0.033	0.409	0.167	-0.834	0.768	-0.082	0.935
D (7:0 - 17:11)	Hashim 2014/Patel 2014	post-surgery	norms	WASI: FSIQ	10.04 yrs	0.521	0.122	0.015	0.283	0.759	4.285	0.000
D (7:0 - 17:11)	ILP D	post-surgery	peers	WISC-IV: FSIQ	7 yrs	-0.290	0.137	0.019	-0.558	-0.022	-2.122	0.034
D (7:0 - 17:11)	Kljajic 2019/2020	post-surgery	norms	WISC-IV: FSIQ	11.5 yrs	-0.254	0.158	0.025	-0.563	0.055	-1.610	0.107
D (7:0 - 17:11)						0.016	0.190	0.036	-0.357	0.388	0.083	0.934

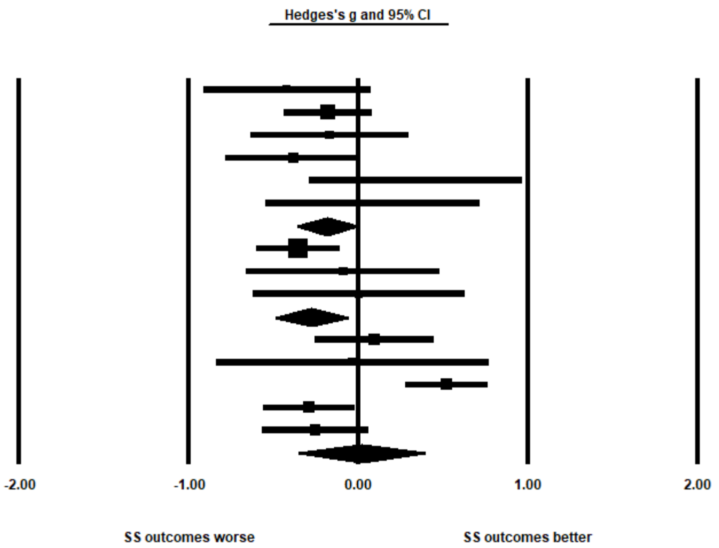

3d. Motor functioning: conservatively managed samples

Group by Age category	Study name	Surgical status	Comparison type	Measure	Age at assessment	Hedges's g	Standard error	Variance	Lower limit	Upper limit	Z-Value	p-Value
B (1:0 - 2:11)	Bellew 2005/2011/2015/2019 B	CM	norms	Combined	Combined	-0.152	0.278	0.077	-0.697	0.394	-0.545	0.586
B (1:0 - 2:11)						-0.152	0.278	0.077	-0.697	0.394	-0.545	0.586

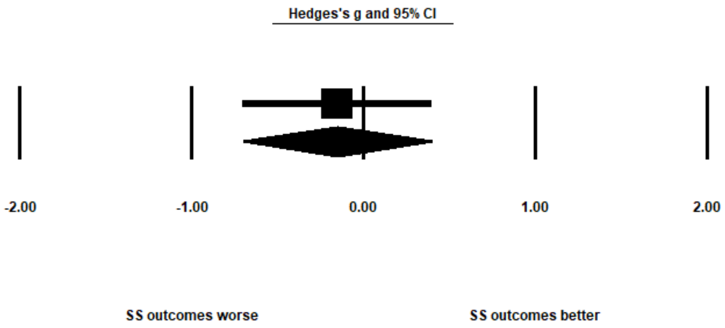

© 2021 Osborn AJ et al. *JAMA Network Open.*

3e. Motor functioning: pre-surgical samples

Group by Age category	Study name	Surgical status	Comparison type	Measure	Age at assessment	Hedges's g	Standard error	Variance	Lower limit	Upper limit	Z-Value	p-Value
						Statistics for each study						
A (0 - 0:11)	Bellew 2005/2011/2015/2019 A	pre-surgery	peers	Combined	6.9 mths	-0.436	0.268	0.072	-0.961	0.090	-1.626	0.104
A (0 - 0:11)	Da Costa 2012/2013	pre-surgery	norms	BSID-II PDI	8.2 mths	-0.822	0.198	0.039	-1.210	-0.434	-4.153	0.000
A (0 - 0:11)	Gewalli 2001	pre-surgery	norms	Combined	6.9 mths	0.442	0.201	0.040	0.049	0.836	2.202	0.028
A (0 - 0:11)	ILP A	pre-surgery	peers	BSID-II PDI	7 mths	-0.409	0.156	0.024	-0.715	-0.103	-2.621	0.009
A (0 - 0:11)	Imahiyerobo 2019	pre-surgery	norms	Combined	5.1 mths	0.211	0.117	0.014	-0.018	0.439	1.808	0.071
A (0 - 0:11)	Speltz 1997	pre-surgery	peers	BSID I PDI	4.1 mths	-0.467	0.326	0.107	-1.107	0.173	-1.431	0.153
A (0 - 0:11)						-0.228	0.205	0.042	-0.630	0.174	-1.113	0.266
B (1:0 - 2:11)	Lee 2015/2017	pre-surgery	norms	BSID-II PDI	17.5 mths	-1.037	0.167	0.028	-1.365	-0.710	-6.209	0.000
B (1:0 - 2:11)						-1.037	0.167	0.028	-1.365	-0.710	-6.209	0.000

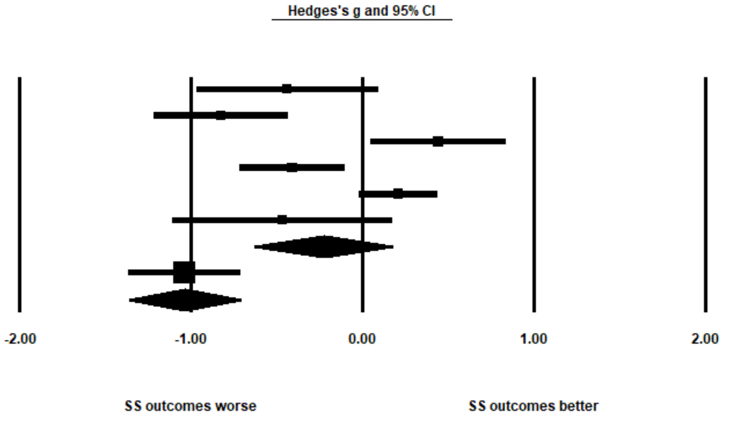

3f. Motor functioning: post-surgical samples

Group by Age category	Study name	Surgical status	Comparison type	Measure	Age at assessment	Hedges's g	Standard error	Variance	Lower limit	Upper limit	Z-Value	p-Value
						Statistics for each study						
B (1:0 - 2:11)	Bellew 2005/2011/2015/2019 B	post-surgery	norms	Combined	15.2 mths	0.406	0.148	0.022	0.117	0.696	2.750	0.006
B (1:0 - 2:11)	Da Costa 2012/2013	post-surgery	norms	BSID-II PDI	21.2 mths	-0.887	0.252	0.063	-1.380	-0.394	-3.528	0.000
B (1:0 - 2:11)	Gewalli 2001	post-surgery	norms	Combined	16.3 mths	0.162	0.200	0.040	-0.230	0.555	0.810	0.418
B (1:0 - 2:11)	ILP B	post-surgery	peers	BSID-II PDI	18 mths	-0.225	0.132	0.017	-0.483	0.033	-1.710	0.087
B (1:0 - 2:11)	Lee 2015/2017	post-surgery	norms	BSID-II PDI	31.5 mths	-0.405	0.201	0.041	-0.800	-0.010	-2.009	0.045
B (1:0 - 2:11)	Speltz 1997	post-surgery	peers	BSID I PDI	Combined	0.000	0.320	0.102	-0.627	0.627	0.000	1.000
B (1:0 - 2:11)						-0.143	0.182	0.033	-0.499	0.213	-0.786	0.432
C (3:0 - 6:11)	Bellew 2005/2011/2015/2019 C	post-surgery	norms	Combined	5 yrs	0.744	0.143	0.021	0.463	1.026	5.188	0.000
C (3:0 - 6:11)	ILP C	post-surgery	peers	BSID-II PDI	3 yrs	-0.313	0.124	0.015	-0.556	-0.071	-2.534	0.011
C (3:0 - 6:11)	Moreno-Villagomez 2020	post-surgery	norms	WPPSI-III: motor	54.2 mths	-0.139	0.317	0.101	-0.761	0.482	-0.440	0.660
C (3:0 - 6:11)						0.108	0.397	0.157	-0.669	0.885	0.272	0.785
D (7:0 - 17:11)	Hashim 2014 / Patel 2014	post-surgery	norms	Beery VMI	10.04 yrs	0.265	0.124	0.015	0.022	0.507	2.141	0.032
D (7:0 - 17:11)	ILP D	post-surgery	peers	Combined	7 yrs	-0.209	0.140	0.020	-0.484	0.066	-1.488	0.137
D (7:0 - 17:11)	Virtanen 1999	post-surgery	peers	Combined	146.8 mths	-0.148	0.327	0.107	-0.789	0.492	-0.454	0.650
D (7:0 - 17:11)						-0.002	0.185	0.034	-0.365	0.361	-0.010	0.992

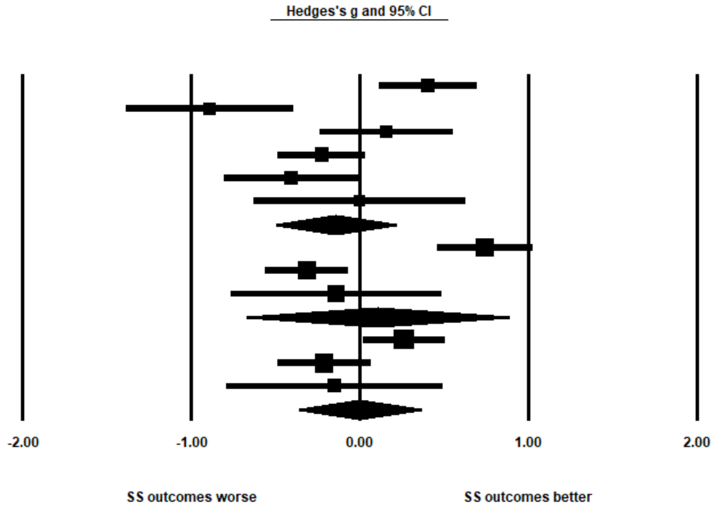

© 2021 Osborn AJ et al. *JAMA Network Open.*

3g. Verbal ability / language: conservatively managed samples

Group by Age category	Study name	Surgical status	Measure	Comparison type	Age at assessment	Hedges's g	Standard error	Variance	Lower limit	Upper limit	Z-Value	p-Value
B (1:0 - 2:11)	Bellew 2005/2011/2015/2019 B	CM	GMDS: hearing & speech	norms	Combined	-0.043	0.278	0.077	-0.588	0.502	-0.155	0.877
B (1:0 - 2:11)						-0.043	0.278	0.077	-0.588	0.502	-0.155	0.877
D (7:0 - 17:11)	Bellew 2005/2011/2015/2019 D	CM	WISC-IV: VIQ	norms	10 yrs	0.227	0.268	0.072	-0.299	0.752	0.845	0.398
D (7:0 - 17:11)						0.227	0.268	0.072	-0.299	0.752	0.845	0.398

Hedges's g and 95% CI

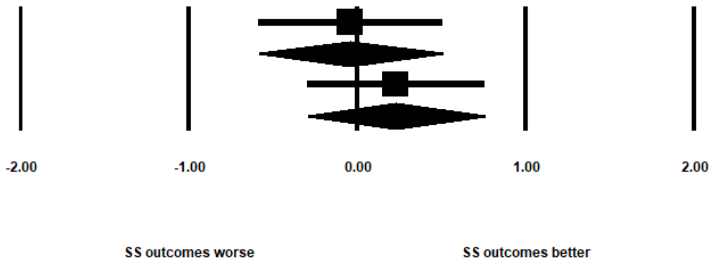

3h. Verbal ability / language: pre-surgical samples

Group by Age category	Study name	Surgical status	Measure	Comparison type	Age at assessment	Hedges's g	Standard error	Variance	Lower limit	Upper limit	Z-Value	p-Value
A (0 - 0:11)	Bellew 2005/2011/2015/2019 A	pre-surgery	GMDS: hearing & speech	peers	6.9 mths	-0.013	0.264	0.069	-0.530	0.503	-0.050	0.961
A (0 - 0:11)	Chuang 2018	pre-surgery	BSID: language score	peers	133 days	0.321	0.420	0.176	-0.502	1.144	0.764	0.445
A (0 - 0:11)	Gewalli 2001	pre-surgery	GMDS: hearing & speech	norms	6.9 mths	0.240	0.200	0.040	-0.152	0.633	1.199	0.230
A (0 - 0:11)	ILP A	pre-surgery	Combined	peers	6 mths	-0.029	0.155	0.024	-0.332	0.274	-0.189	0.850
A (0 - 0:11)	Imahiyerobo 2019	pre-surgery	Combined	norms	5.1 mths	0.145	0.116	0.014	-0.084	0.373	1.241	0.215
A (0 - 0:11)						0.106	0.079	0.006	-0.048	0.261	1.346	0.178

Hedges's g and 95% CI

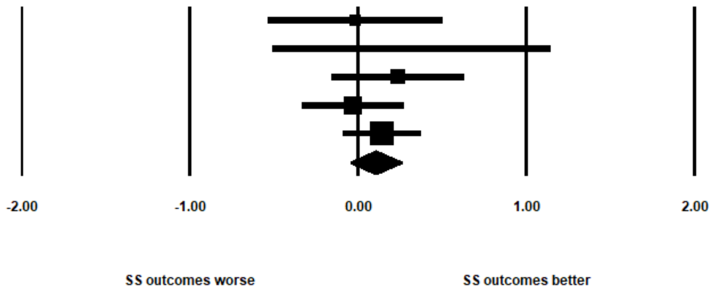

© 2021 Osborn AJ et al. *JAMA Network Open.*

3i. Verbal ability / language: post-surgical samples

Group by Age category	Study name	Surgical status	Measure	Comparison type	Age at assessment	Hedges's g	Standard error	Variance	Lower limit	Upper limit	Z-Value	p-Value
B (1:0 - 2:11)	Bellew 2005/2011/2015/2019 B	post-surgery	GMDS: hearing & speech	norms	15.2 mths	0.231	0.147	0.022	-0.058	0.520	1.570	0.116
B (1:0 - 2:11)	Gewalli 2001	post-surgery	GMDS: hearing & speech	norms	16.3 mths	0.020	0.200	0.040	-0.373	0.412	0.099	0.921
B (1:0 - 2:11)	ILP B	post-surgery	Combined	peers	18 mths	-0.219	0.132	0.017	-0.477	0.039	-1.663	0.096
B (1:0 - 2:11)						0.004	0.147	0.022	-0.284	0.291	0.026	0.979
C (3:0 - 6:11)	Bellew 2005/2011/2015/2019 C	post-surgery	GMDS: hearing & speech	norms	5 yrs	1.103	0.144	0.021	0.821	1.385	7.670	0.000
C (3:0 - 6:11)	ILP C	post-surgery	Combined	peers	36 mths	-0.437	0.125	0.016	-0.683	-0.192	-3.491	0.000
C (3:0 - 6:11)	Korpilahti 2012	post-surgery	Combined	norms	3.4 yrs	0.094	0.238	0.056	-0.372	0.560	0.395	0.693
C (3:0 - 6:11)	Moreno-Villagomez 2020	post-surgery	Combined	norms	54.2 mths	-0.146	0.317	0.101	-0.768	0.475	-0.461	0.645
C (3:0 - 6:11)						0.161	0.432	0.187	-0.686	1.008	0.372	0.710
D (7:0 - 17:11)	Bellew 2005/2011/2015/2019 D	post-surgery	WISC-IV: VIQ	norms	Combined	0.355	0.177	0.031	0.007	0.702	2.000	0.046
D (7:0 - 17:11)	Cabrejo 2019	post-surgery	WISC-III: VIQ	peers	12.1 yrs	-1.197	0.468	0.219	-2.115	-0.279	-2.557	0.011
D (7:0 - 17:11)	Hashim 2014/Patel 2014	post-surgery	WASI: VIQ	norms	10.5 yrs	0.459	0.121	0.015	0.221	0.697	3.778	0.000
D (7:0 - 17:11)	ILP D	post-surgery	Combined	peers	7 yrs	-0.104	0.138	0.019	-0.374	0.165	-0.757	0.449
D (7:0 - 17:11)	Kljajic 2019/2020	post-surgery	WISC-IV: VCI	norms	11.5 yrs	-0.160	0.158	0.025	-0.469	0.149	-1.015	0.310
D (7:0 - 17:11)	Virtanen 1999	post-surgery	Combined	peers	146.8 mths	-0.897	0.343	0.117	-1.569	-0.225	-2.617	0.009
D (7:0 - 17:11)						-0.124	0.193	0.037	-0.504	0.255	-0.642	0.521

Statistics for each study

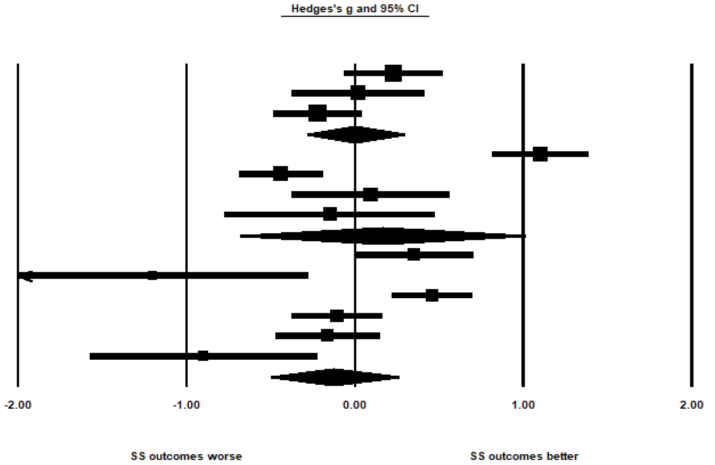

3j. Visuospatial ability / language: conservatively managed samples

Group by Age category	Study name	Surgical status	Measure	Type of comparison	Age at assessment	Hedges's g	Standard error	Variance	Lower limit	Upper limit	Z-Value	p-Value
B (1:0 - 2:11)	Bellew 2005/2011/2015/2019 B	CM	GMDS: performance	norms	Combined	0.086	0.278	0.077	-0.459	0.631	0.311	0.756
B (1:0 - 2:11)						0.086	0.278	0.077	-0.459	0.631	0.311	0.756
D (7:0 - 17:11)	Bellew 2005/2011/2015/2019 D	CM	WISC-IV: PIQ	norms	10 yrs	-0.040	0.268	0.072	-0.565	0.485	-0.149	0.881
D (7:0 - 17:11)						-0.040	0.268	0.072	-0.565	0.485	-0.149	0.881

Statistics for each study

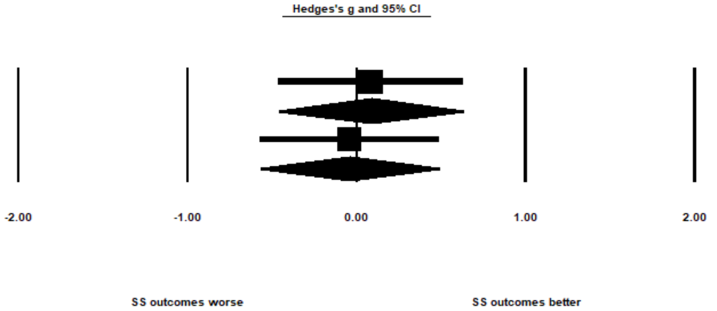

© 2021 Osborn AJ et al. *JAMA Network Open.*

3k. Visuospatial ability / language: pre-surgical samples

Group by Age category	Study name	Surgical status	Measure	Type of comparison	Age at assessment	Hedges's g	Standard error	Variance	Lower limit	Upper limit	Z-Value	p-Value
						Statistics for each study						
A (0 - 0:11)	Bellew 2005/2011/2015/2019 A	pre-surgery	GMDS: performance	peers	6.9 mths	-0.090	0.264	0.070	-0.607	0.427	-0.341	0.733
A (0 - 0:11)	Gewalli 2001	pre-surgery	GMDS: performance	norms	6.9 mths	0.438	0.201	0.040	0.044	0.831	2.181	0.029
A (0 - 0:11)						0.202	0.262	0.069	-0.313	0.716	0.768	0.442

Hedges's g and 95% CI

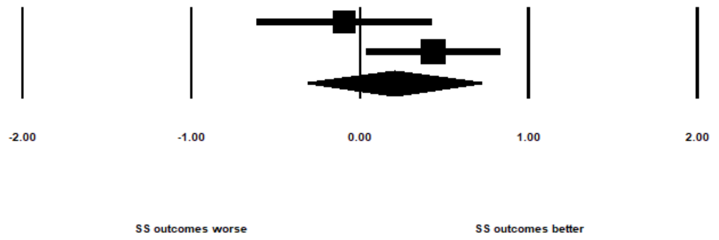

3l. Visuospatial ability / language: post-surgical samples

Group by Age category	Study name	Surgical status	Measure	Type of comparison	Age at assessment	Hedges's g	Standard error	Variance	Lower limit	Upper limit	Z-Value	p-Value
						Statistics for each study						
B (1:0 - 2:11)	Bellew 2005/2011/2015/2019 B	post-surgery	WISC-IV: performance	norms	15 mths	0.492	0.143	0.020	0.211	0.772	3.434	0.001
B (1:0 - 2:11)	Gewalli 2001	post-surgery	GMDS: performance	norms	16.3 mths	0.354	0.201	0.040	-0.039	0.747	1.763	0.078
B (1:0 - 2:11)						0.445	0.117	0.014	0.217	0.673	3.819	0.000
C (3:0 - 6:11)	Bellew 2005/2011/2015/2019 C	post-surgery	WISC-IV: performance	norms	60 mths	0.385	0.143	0.020	0.105	0.666	2.692	0.007
C (3:0 - 6:11)	Moreno-Villagomez 2020	post-surgery	WPPSI-III: PIQ	norms	54.2 mths	-0.033	0.317	0.101	-0.655	0.588	-0.105	0.916
C (3:0 - 6:11)						0.271	0.186	0.035	-0.093	0.636	1.458	0.145
D (7:0 - 17:11)	Bellew 2005/2011/2015/2019 D	post-surgery	WISC-IV: PIQ	norms	Combined	-0.186	0.177	0.031	-0.533	0.162	-1.047	0.295
D (7:0 - 17:11)	Cabrejo 2019	post-surgery	WISC-III: PIQ	peers	12.1 yrs	-0.319	0.431	0.186	-1.165	0.526	-0.740	0.459
D (7:0 - 17:11)	Hashim 2014/Patel 2014	post-surgery	WASI: PIQ	norms	10.04 yrs	0.486	0.121	0.015	0.248	0.724	3.999	0.000
D (7:0 - 17:11)	ILP D	post-surgery	Combined	peers	7 yrs	0.018	0.140	0.020	-0.256	0.293	0.131	0.896
D (7:0 - 17:11)	Kljajic 2019/2020	post-surgery	WISC-IV: PRI	norms	11.5 yrs	0.261	0.158	0.025	-0.048	0.570	1.656	0.098
D (7:0 - 17:11)	Virtanen 1999	post-surgery	WISC-R: block design	peers	146.8 mths	-0.430	0.330	0.109	-1.077	0.216	-1.304	0.192
D (7:0 - 17:11)						0.058	0.141	0.020	-0.219	0.334	0.407	0.684

Hedges's g and 95% CI

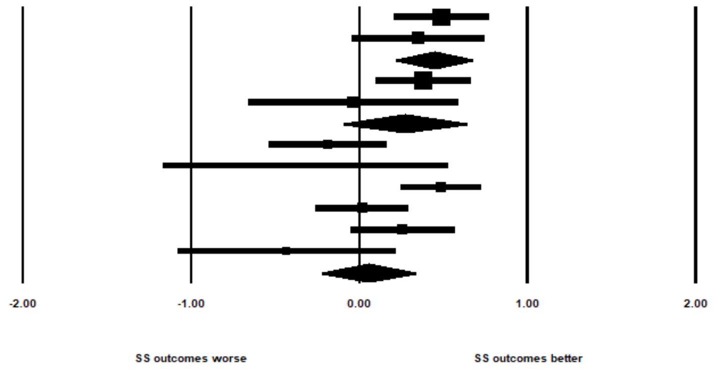

© 2021 Osborn AJ et al. *JAMA Network Open.*

3m. Adaptive & social skills: conservatively managed samples

Group by Age category	Study name	Surgical status	Comparison type	Measure	Age at assessment	Hedges's g	Standard error	Variance	Lower limit	Upper limit	Z-Value	p-Value
B (1:0 - 2:11)	Bellew 2005/2011/2015/2019 B	CM	norms	GMDS I personal-social	Combined	-0.140	0.278	0.077	-0.685	0.405	-0.502	0.616
B (1:0 - 2:11)						-0.140	0.278	0.077	-0.685	0.405	-0.502	0.616

Statistics for each study

Hedges's g and 95% CI

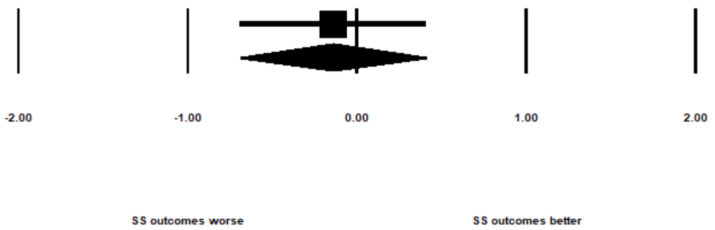

3n. Adaptive & social skills: pre-surgical samples

Group by Age category	Study name	Surgical status	Comparison type	Measure	Age at assessment	Hedges's g	Standard error	Variance	Lower limit	Upper limit	Z-Value	p-Value
A (0 - 0:11)	Bellew 2005/2011/2015/2019 A	pre-surgery	peers	GMDS I personal-social	6.9 mths	-0.206	0.264	0.070	-0.724	0.312	-0.781	0.435
A (0 - 0:11)	Gewalli 2001	pre-surgery	norms	GMDS I personal-social	6.9 mths	-0.209	0.200	0.040	-0.602	0.184	-1.043	0.297
A (0 - 0:11)						-0.208	0.160	0.025	-0.521	0.105	-1.303	0.193

Statistics for each study

Hedges's g and 95% CI

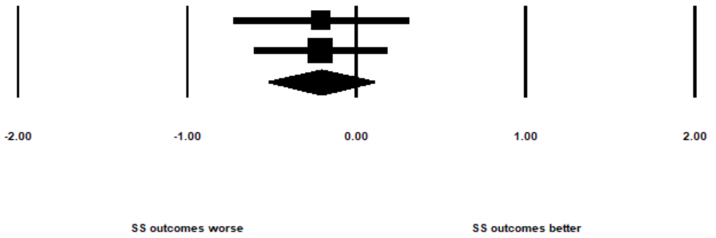

3o. Adaptive & social skills: post-surgical samples

Group by Age category	Study name	Surgical status	Comparison type	Measure	Age at assessment	Hedges's g	Standard error	Variance	Lower limit	Upper limit	Z-Value	p-Value
B (1:0 - 2:11)	Bellew 2005/2011/2015/2019 B	post-surgery	norms	GMDS I personal-social	15.2 mths	0.151	0.147	0.022	-0.138	0.440	1.024	0.306
B (1:0 - 2:11)	Gewalli 2001	post-surgery	norms	GMDS I personal-social	15.2 mths	-0.127	0.200	0.040	-0.520	0.265	-0.635	0.526
B (1:0 - 2:11)						0.045	0.135	0.018	-0.220	0.310	0.333	0.739
C (3:0 - 6:11)	Bellew 2005/2011/2015/2019 C	post-surgery	norms	GMDS I personal-social	5 yrs	0.285	0.143	0.020	0.005	0.565	1.992	0.046
C (3:0 - 6:11)						0.285	0.143	0.020	0.005	0.565	1.992	0.046

Statistics for each study

Hedges's g and 95% CI

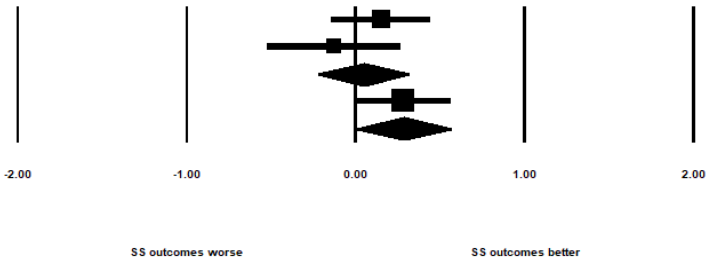

© 2021 Osborn AJ et al. *JAMA Network Open.*

3p. Arithmetic: post-surgical samples

Group by Age category	Study name	Surgical status	Comparison type	Measure	Age	Hedges's g	Standard error	Variance	Lower limit	Upper limit	Z-Value	p-Value
C (3:0 - 6:11)	Moreno-Villagomez 2020	post-surgery	norms	McCarthy Scales: quantitative index	54.2 mths	-0.239	0.318	0.101	-0.862	0.383	-0.753	0.451
C (3:0 - 6:11)						-0.239	0.318	0.101	-0.862	0.383	-0.753	0.451
D (7:0 - 17:11)	ILP D	post-surgery	peers	WRAT-4: math computation	7 yrs	-0.212	0.137	0.019	-0.481	0.057	-1.547	0.122
D (7:0 - 17:11)						-0.212	0.137	0.019	-0.481	0.057	-1.547	0.122

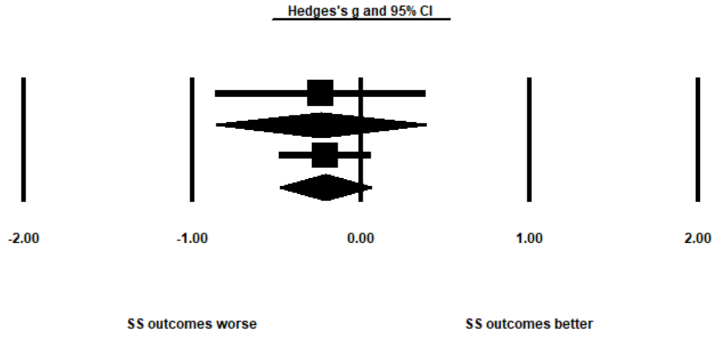

3q. Attention: post-surgical samples

Group by Age category	Study name	Surgical status	Comparison type	Measure	Age at assessment	Hedges's g	Standard error	Variance	Lower limit	Upper limit	Z-Value	p-Value
D (7:0 - 17:11)	ILP D	post-surgery	peers	Combined	7 yrs	-0.108	0.137	0.019	-0.376	0.160	-0.788	0.431
D (7:0 - 17:11)	Kljajic 2020	post-surgery	norms	Combined	11.4 yrs	-0.512	0.165	0.027	-0.835	-0.190	-3.111	0.002
D (7:0 - 17:11)	Virtanen 1999	post-surgery	peers	WISC-R: digit span	146.8 mths	-0.821	0.340	0.116	-1.487	-0.154	-2.414	0.016
D (7:0 - 17:11)						-0.405	0.193	0.037	-0.784	-0.026	-2.095	0.036

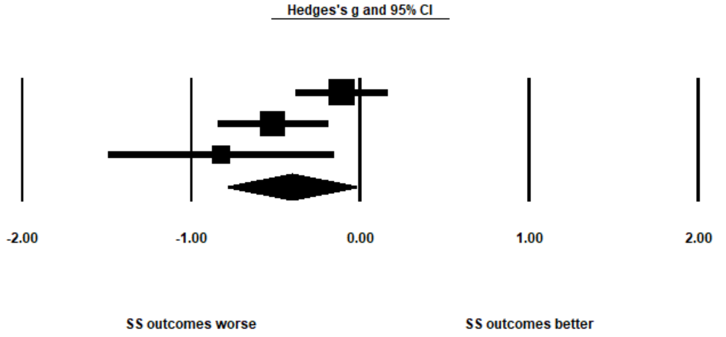

© 2021 Osborn AJ et al. *JAMA Network Open.*

3r. Executive Function: post-surgical samples

Group by Age category	Study name	Surgical status	Comparison type	Measure	Age at assessment	Hedges's g	Standard error	Variance	Lower limit	Upper limit	Z-Value	p-Value
D (7:0 - 16:11)	ILP	post-surgery	peers	Combined	7 yrs	-0.181	0.139	0.019	-0.453	0.091	-1.307	0.191
D (7:0 - 16:11)						-0.181	0.139	0.019	-0.453	0.091	-1.307	0.191

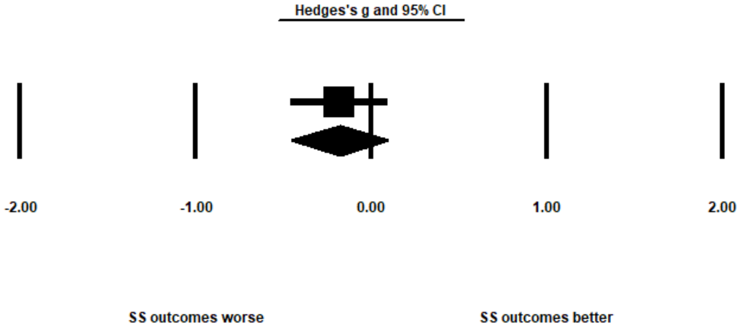

3s. Global development: conservatively managed samples

Group by Age category	Study name	Surgical status	Comparison type	Measure	Age at assessment	Hedges's g	Standard error	Variance	Lower limit	Upper limit	Z-Value	p-Value
B (1:0 - 2:11)	Bellew 2005/2011/2015/2019 B	CM	norms	GMDS: GQ	15.6 mths	0.444	0.213	0.045	0.027	0.861	2.088	0.037
B (1:0 - 2:11)						0.444	0.213	0.045	0.027	0.861	2.088	0.037
C (3:0 - 6:11)	Bellew 2005/2011/2015/2019 C	CM	norms	GMDS: GQ	42.9 mths	0.719	0.210	0.044	0.308	1.131	3.430	0.001
C (3:0 - 6:11)						0.719	0.210	0.044	0.308	1.131	3.430	0.001

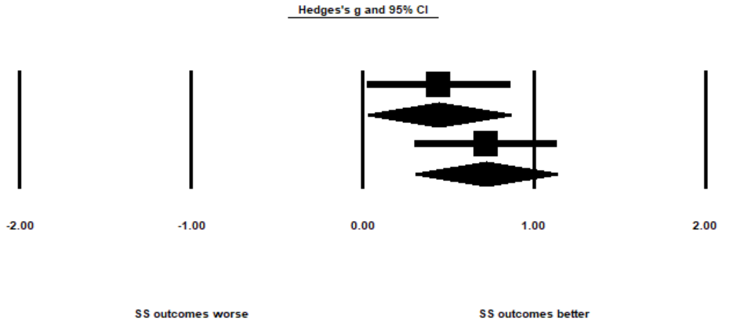

3t. Global development: pre-surgical samples

Group by Age category	Study name	Surgical status	Comparison type	Measure	Age at assessment	Hedges's g	Standard error	Variance	Lower limit	Upper limit	Z-Value	p-Value
A (0 - 0:11)	Bellew 2005/2011/2015/2019 A	pre-surgery	peers	GMDS: GQ	6.9 mths	-0.305	0.265	0.070	-0.825	0.214	-1.151	0.250
A (0 - 0:11)	Gewalli 2001	pre-surgery	norms	GMDS: GDQ	6.9 mths	0.302	0.200	0.040	-0.091	0.695	1.505	0.132
A (0 - 0:11)						0.023	0.302	0.091	-0.570	0.616	0.076	0.939

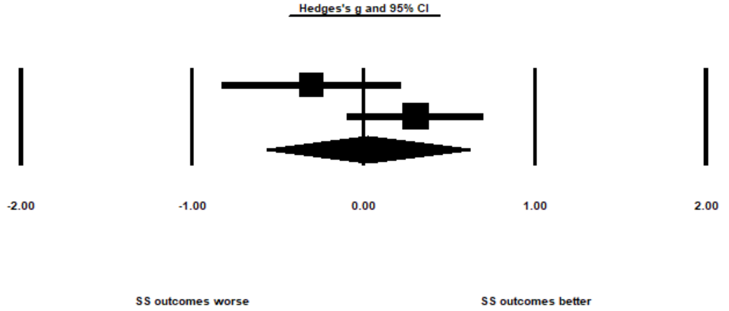

© 2021 Osborn AJ et al. *JAMA Network Open.*

3u. Global development: post-surgical samples

Group by Age category	Study name	Surgical status	Comparison type	Measure	Age at assessment	Hedges's g	Standard error	Variance	Lower limit	Upper limit	Z-Value	p-Value
B (1:0 - 2:11)	Bellew 2005/2011/2015/2019 B	post-surgery	norms	GMDS: GQ	15.2 mths	0.386	0.148	0.022	0.096	0.675	2.611	0.009
B (1:0 - 2:11)	Gewalli 2001	post-surgery	norms	GMDS GDQ	16.3 mths	0.094	0.200	0.040	-0.299	0.486	0.467	0.640
B (1:0 - 2:11)						0.271	0.143	0.020	-0.009	0.551	1.899	0.058
C (3:0 - 6:11)	Bellew 2005/2011/2015/2019 C	post-surgery	norms	GMDS: GQ	5 yrs age	0.679	0.143	0.021	0.398	0.960	4.740	0.000
C (3:0 - 6:11)						0.679	0.143	0.021	0.398	0.960	4.740	0.000

Statistics for each study; Hedges's g and 95% CI

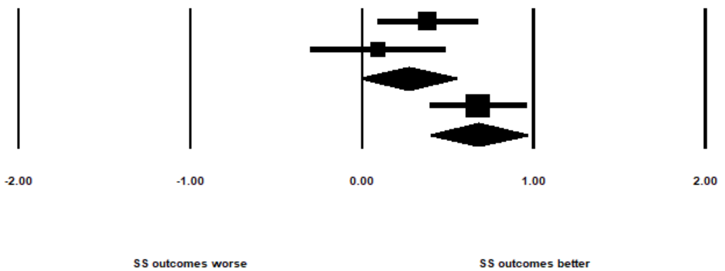

3v. Memory – longer-term: post-surgical samples

Group by Age category	Study name	Surgical status	Comparison type	Measure	Age at assessment	Hedges's g	Standard error	Variance	Lower limit	Upper limit	Z-Value	p-Value
D (7:0 - 17:11)	ILP	post-surgery	peers	Combined	7 yrs	-0.177	0.139	0.019	-0.450	0.095	-1.276	0.202
D (7:0 - 17:11)						-0.177	0.139	0.019	-0.450	0.095	-1.276	0.202

Statistics for each study; Hedges's g and 95% CI

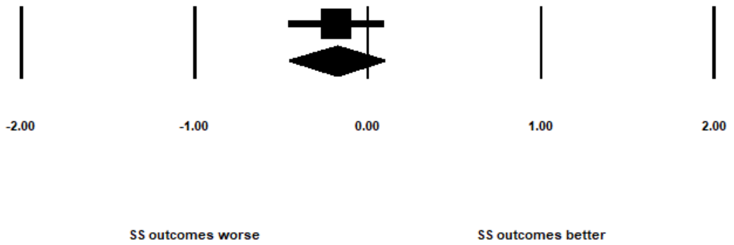

3w. Memory – shorter-term: post-surgical samples

Group by Age category	Study name	Surgical status	Comparison group	Measure	Age at assessment	Hedges's g	Standard error	Variance	Lower limit	Upper limit	Z-Value	p-Value
C (3:0 - 6:11)	Moreno-Villagomez 2020	post-surgery	norms	MSCA: Memory	54.2 mths	-0.279	0.317	0.101	-0.901	0.342	-0.880	0.379
C (3:0 - 6:11)						-0.279	0.317	0.101	-0.901	0.342	-0.880	0.379
D (7:0 - 17:11)	ILP	post-surgery	peers	Combined	7 yrs	-0.235	0.138	0.019	-0.506	0.036	-1.697	0.090
D (7:0 - 17:11)	Kljajic 2019/2020	post-surgery	norms	WISC-IV: WMI	11.5 yrs	-0.488	0.158	0.025	-0.797	-0.179	-3.095	0.002
D (7:0 - 17:11)	Virtanen 1999	post-surgery	peers	Bentons Visual Retention	146.8 mths	0.000	0.326	0.106	-0.639	0.639	0.000	1.000
D (7:0 - 17:11)						-0.309	0.115	0.013	-0.534	-0.084	-2.691	0.007

Statistics for each study; Hedges's g and 95% CI

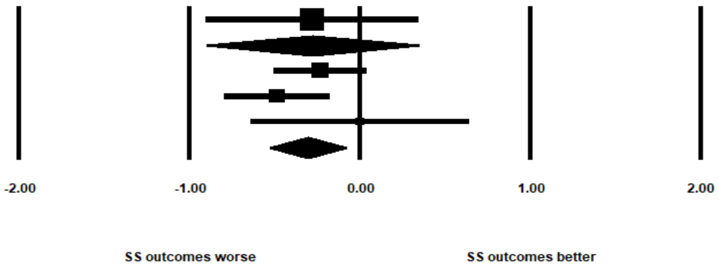

© 2021 Osborn AJ et al. *JAMA Network Open.*

3x. Processing speed: post-surgical samples

Group by Age category	Study name	Surgical status	Comparison type	Measure	Age at assessment	Hedges's g	Standard error	Variance	Lower limit	Upper limit	Z-Value	p-Value
C (3:0 - 6:11)	Moreno-Villagomez 2020	post-surgery	norms	WPPSI-III: Processing speed quotient	54.2 mths	0.267	0.379	0.143	-0.475	1.009	0.704	0.481
C (3:0 - 6:11)						0.267	0.379	0.143	-0.475	1.009	0.704	0.481
D (7:0 - 17:11)	Kljajic 2019/2020	post-surgery	norms	WISC-IV:PSI	11.5 yrs	-0.648	0.158	0.025	-0.957	-0.339	-4.104	0.000
D (7:0 - 17:11)						-0.648	0.158	0.025	-0.957	-0.339	-4.104	0.000

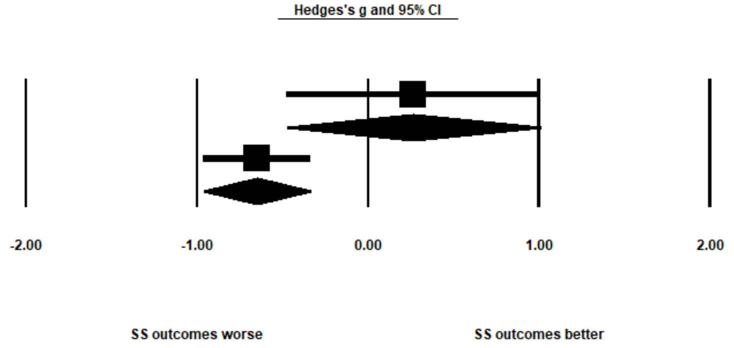

© 2021 Osborn AJ et al. *JAMA Network Open.*

SUBJECTIVE MEASURES

3y. Behavior - externalizing: conservatively managed samples

Group by Age category	Study name	Surgical status	Comparison type	Measure	Age at assessment	Hedges's g	Standard error	Variance	Lower limit	Upper limit	Z-Value	p-Value
D (7:0 - 17:11)	Boltshauser 2003	CM	norms	CBCL: externalising	9.25 yrs	-0.250	0.205	0.042	-0.651	0.152	-1.219	0.223
D (7:0 - 17:11)						-0.250	0.205	0.042	-0.651	0.152	-1.219	0.223

Statistics for each study: Hedges's g through p-Value columns.

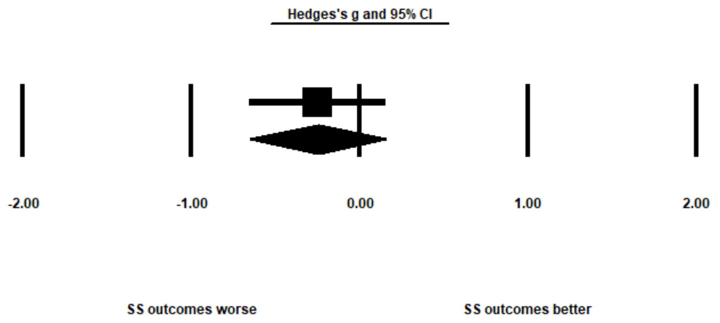

3z. Behavior - externalizing: post-surgical samples

Group by Age category	Study name	Surgical status	Comparison type	Measure	Age at assessment	Hedges's g	Standard error	Variance	Lower limit	Upper limit	Z-Value	p-Value
C (3:0 - 6:11)	Speltz 1993	post-surgery	peers	Combined	70 mths	-0.388	0.479	0.230	-1.327	0.551	-0.809	0.418
C (3:0 - 6:11)						-0.388	0.479	0.230	-1.327	0.551	-0.809	0.418
D (7:0 - 17:11)	Chandler 2020	post-surgery	norms	BASC: externalizing	10.3 yrs	0.004	0.116	0.014	-0.224	0.232	0.034	0.973
D (7:0 - 17:11)	Hashim 2014 / Patel 2014	post-surgery	norms	BASC 2: externalising	10.04 yrs	-0.222	0.120	0.014	-0.458	0.014	-1.842	0.065
D (7:0 - 17:11)	ILP	post-surgery	peers	Combined	7 yrs	-0.013	0.136	0.019	-0.280	0.255	-0.092	0.927
D (7:0 - 17:11)						-0.080	0.074	0.005	-0.225	0.066	-1.075	0.282

Statistics for each study: Hedges's g through p-Value columns.

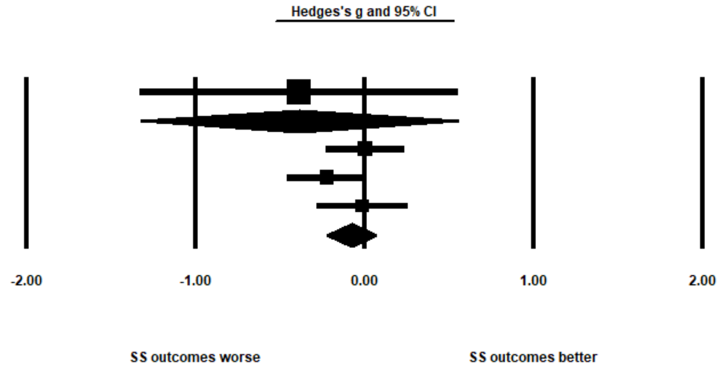

© 2021 Osborn AJ et al. *JAMA Network Open.*

3aa. Behavior - internalizing: conservatively managed samples

Group by Age category	Study name	Surgical status	Comparison type	Measure	Age at assessment	Hedges's g	Standard error	Variance	Lower limit	Upper limit	Z-Value	p-Value
D (7:0 - 17:11)	Boltshauser 2003	CM	norms	CBCL: internalizing	9.25 yrs	-0.300	0.205	0.042	-0.702	0.101	-1.465	0.143
D (7:0 - 17:11)						-0.300	0.205	0.042	-0.702	0.101	-1.465	0.143

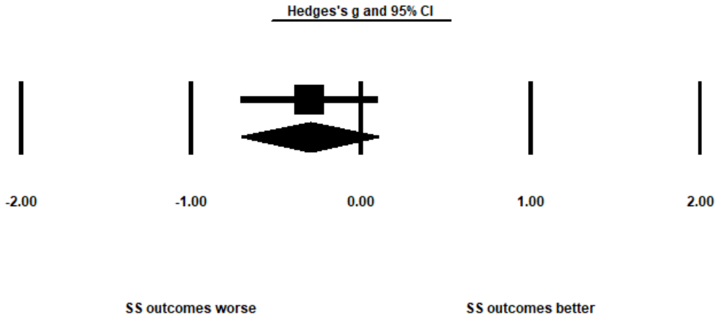

3ab. Behavior - internalizing: post-surgical samples

Group by Age category	Study name	Surgical status	Comparison type	Measure	Age at assessment	Hedges's g	Standard error	Variance	Lower limit	Upper limit	Z-Value	p-Value
C (3:0 - 6:11)	Speltz 1993	post-surgery	peers	Combined	70 mths	-0.662	0.485	0.235	-1.613	0.288	-1.366	0.172
C (3:0 - 6:11)						-0.662	0.485	0.235	-1.613	0.288	-1.366	0.172
D (7:0 - 17:11)	Chandler 2020	post-surgery	norms	BASC-2:internalizing	10.3 yrs	-0.105	0.116	0.014	-0.333	0.123	-0.903	0.367
D (7:0 - 17:11)	Hashim 2014/Patel 2014	post-surgery	norms	BASC 2: internalizing	10.04 yrs	-0.056	0.120	0.014	-0.292	0.180	-0.464	0.643
D (7:0 - 17:11)	ILP	post-surgery	peers	Combined	7 yrs	-0.084	0.136	0.019	-0.351	0.184	-0.614	0.539
D (7:0 - 17:11)						-0.082	0.071	0.005	-0.222	0.058	-1.150	0.250

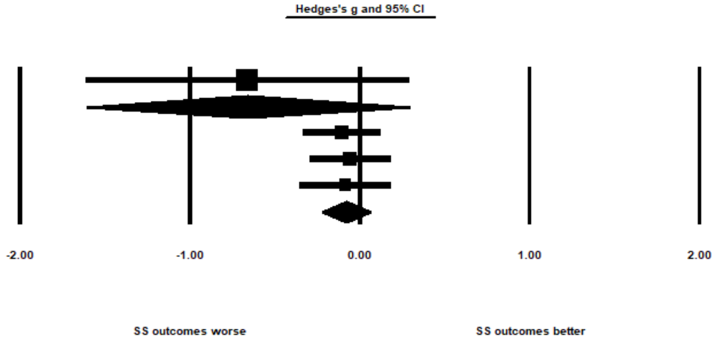

3ac. Behavior - total: conservatively managed samples

Group by Age category	Study name	Surgical status	Comparison type	Measure	Age at assessment	Hedges's g	Standard error	Variance	Lower limit	Upper limit	Z-Value	p-Value
D (7:0 - 17:11)	Boltshauser 2003	CM	norms	CBCL: total score	9.25 yrs	-0.290	0.205	0.042	-0.692	0.112	-1.414	0.157
D (7:0 - 17:11)						-0.290	0.205	0.042	-0.692	0.112	-1.414	0.157

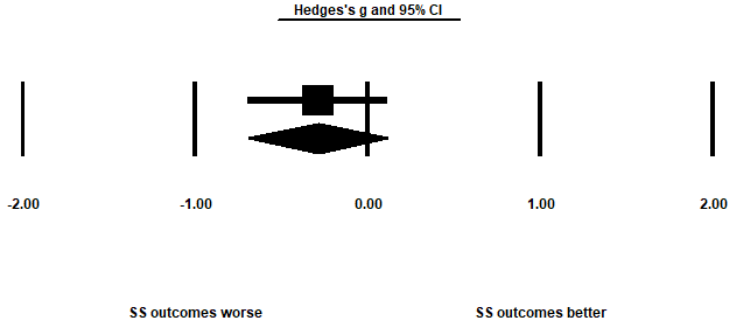

© 2021 Osborn AJ et al. *JAMA Network Open.*

3ad. Behavior - total: post-surgical samples

Group by Age category	Study name	Surgical status	Comparison type	Measure	Age at assessment	Hedges's g	Standard error	Variance	Lower limit	Upper limit	Z-Value	p-Value
C (3:0 - 6:11)	Speltz 1993	post-surgery	peers	Combined	70 mths	0.488	0.481	0.231	-0.455	1.431	1.015	0.310
C (3:0 - 6:11)						0.488	0.481	0.231	-0.455	1.431	1.015	0.310
D (7:0 - 17:11)	Chandler 2020	post-surgery	norms	Combined	10.3 yrs	0.171	0.164	0.027	-0.150	0.493	1.043	0.297
D (7:0 - 17:11)	ILP	post-surgery	peers	Combined	7 yrs	-0.048	0.136	0.019	-0.316	0.219	-0.354	0.723
D (7:0 - 17:11)						0.043	0.108	0.012	-0.169	0.254	0.393	0.694

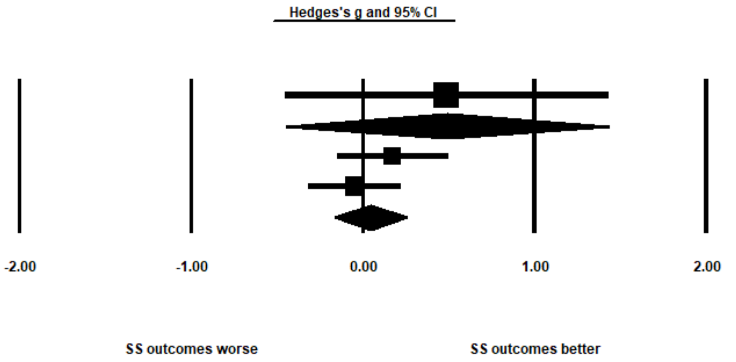

3ae. Executive function: post-surgical samples

Group by Age category	Study name	Surgical status	Comparison type	Measure	Age at assessment	Hedges's g	Standard error	Variance	Lower limit	Upper limit	Z-Value	p-Value
D (7:0 - 16:11)	Chandler 2020	post-surgery	norms	BRIEF- parent GEC	10.3 yrs	-0.175	0.118	0.014	-0.407	0.058	-1.474	0.141
D (7:0 - 16:11)	Hashim 2014/Patel 2014	post-surgery	norms	BRIEF: GEC	10.04 yrs	-0.598	0.123	0.015	-0.839	-0.357	-4.869	0.000
D (7:0 - 16:11)	ILP	post-surgery	peers	Combined	7 yrs	0.039	0.149	0.022	-0.253	0.331	0.264	0.792
D (7:0 - 16:11)						-0.251	0.184	0.034	-0.611	0.110	-1.364	0.173

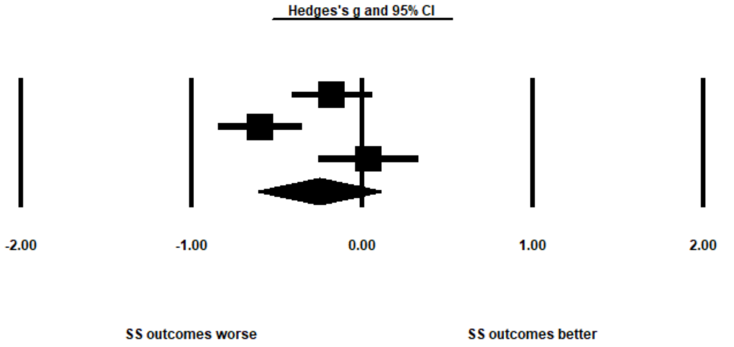

© 2021 Osborn AJ et al. *JAMA Network Open.*

3af. Memory – shorter-term: post-surgical samples

Group by Age category	Study name	Surgical status	Comparison group	Measure	Age at assessment	Hedges's g	Standard error	Variance	Lower limit	Upper limit	Z-Value	p-Value
D (7:0 - 17:11)	Chandler 2020	post-surgery	norms	BRIEF: working memory	10.3 yrs	-0.188	0.118	0.014	-0.420	0.044	-1.588	0.112
D (7:0 - 17:11)	Hashim 2014/Patel 2014	post-surgery	norms	BRIEF: working memory	10.04 yrs	-0.580	0.123	0.015	-0.821	-0.339	-4.720	0.000
D (7:0 - 17:11)						-0.383	0.196	0.038	-0.766	0.001	-1.954	0.051

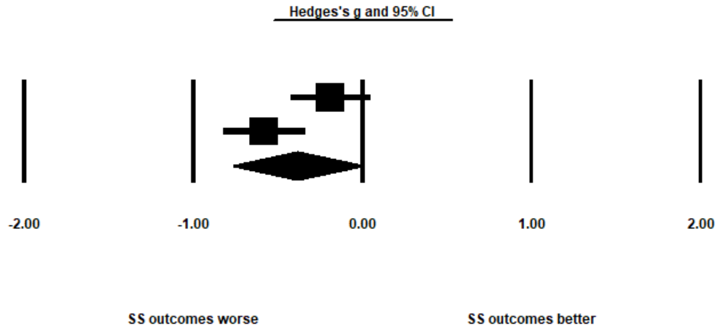

3ag. Quality of life: conservatively managed samples

Group by Age category	Study name	Surgical status	Comparison type	Measure	Age at assessment	Hedges's g	Standard error	Variance	Lower limit	Upper limit	Z-Value	p-Value
D (7:0 - 6:11)	Boltshauser 2003	CM	norms	Combined	9.25 yrs	-0.000	0.205	0.042	-0.403	0.402	-0.001	0.999
D (7:0 - 6:11)						-0.000	0.205	0.042	-0.403	0.402	-0.001	0.999

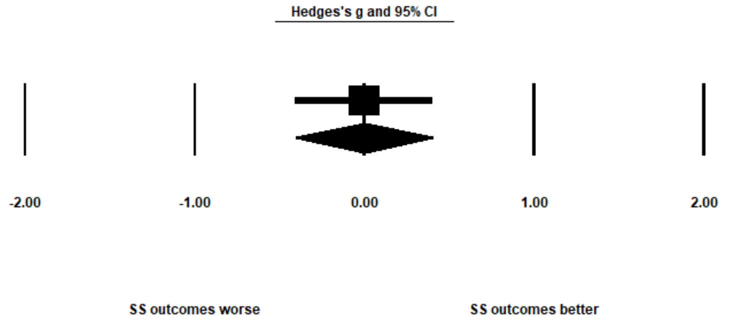

3ah. Quality of life: post-surgical samples

Group by Age category	Study name	Surgical status	Comparison type	Measure	Age at assessment	Hedges's g	Standard error	Variance	Lower limit	Upper limit	Z-Value	p-Value
E (> 18:0)	Mazzaferro 2018	post-surgery	norms	Combined	23 yrs	1.294	0.447	0.200	0.417	2.170	2.892	0.004
E (> 18:0)						1.294	0.447	0.200	0.417	2.170	2.892	0.004

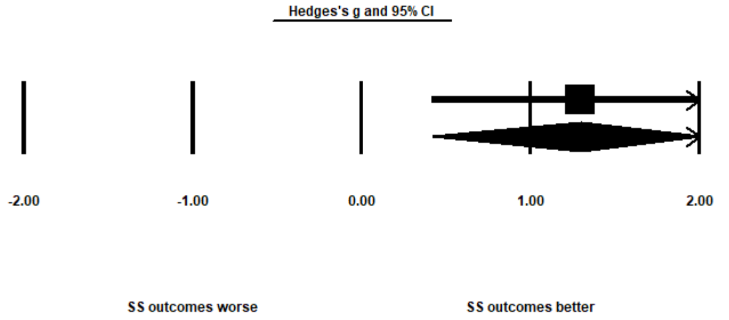

© 2021 Osborn AJ et al. *JAMA Network Open.*

3ai. Satisfaction with appearance: post-surgical samples

Group by Age category	Study name	Surgical status	Comparison type	Measure	Age at assessment	Hedges's g	Standard error	Variance	Lower limit	Upper limit	Z-Value	p-Value
						Statistics for each study						
E (> 18:0)	Salokorpi 2018	post-surgery	peers	100mm visual analog scale	27.4 yrs	-0.061	0.222	0.049	-0.495	0.373	-0.275	0.783
E (> 18:0)						-0.061	0.222	0.049	-0.495	0.373	-0.275	0.783

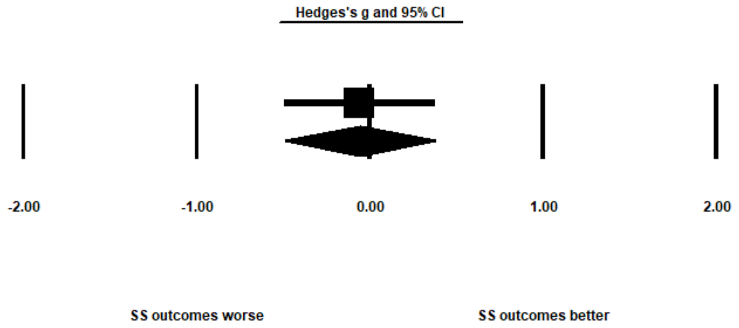

3aj. Self-concept: post-surgical samples

Group by Age category	Study name	Surgical status	Comparison type	Measure	Age at assessment	Hedges's g	Standard error	Variance	Lower limit	Upper limit	Z-Value	p-Value
						Statistics for each study						
C (3:0 - 6:11)	Speltz 1993	post-surgery	peers	Selection of opposing descriptive statements	70 mths	-0.034	0.468	0.219	-0.950	0.883	-0.072	0.943
C (3:0 - 6:11)						-0.034	0.468	0.219	-0.950	0.883	-0.072	0.943

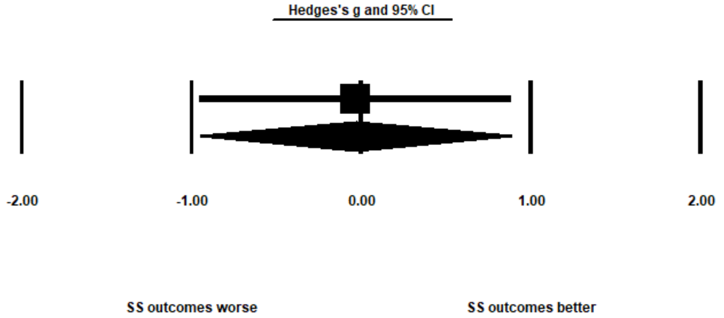

eFigure 3

Pooled analyses for each cognitive, behavioral and psychological domain, partitioned according to surgical status (conservatively managed, presurgical, postsurgical) and age at assessment: A (0 – 0.11); B (1:0 – 2:11); C (3:0 – 6:11); D (7:0 – 17:11); E (>18:0)

Note: ILP = Infant Learning Project

© 2021 Osborn AJ et al. *JAMA Network Open.*

eReferences

1. Arnaud E, Renier D, Marchac D. Prognosis for mental function in scaphocephaly. *Journal of Neurosurgery.* 1995;83(3):476-479.
2. Bellew M, Chumas P, Mueller R, Liddington M, Russell J. Pre- and postoperative developmental attainment in sagittal synostosis. *Archives of Disease in Childhood.* 2005;90(4):346-350.
3. Bellew M, Liddington M, Chumas P, Russell J. Preoperative and postoperative developmental attainment in patients with sagittal synostosis: 5-Year follow-up. *Journal of Neurosurgery: Pediatrics.* 2011;7(2):121-126.
4. Bellew M, Chumas P. Long-term developmental follow-up in children with nonsyndromic craniosynostosis. *Journal of Neurosurgery: Pediatrics.* 2015;16(4):445-451.
5. Bellew M, Mandela RJ, Chumas PD. Impact of age at surgery on neurodevelopmental outcomes in sagittal synostosis. *Journal of Neurosurgery: Pediatrics.* 2019;23(4):434-441.
6. Boltshauser E, Ludwig S, Dietrich F, Landolt MA. Sagittal Craniosynostosis: Cognitive Development, Behaviour, and Quality of Life in Unoperated Children. *Neuropediatrics.* 2003;34(6):293-300.
7. Byun IH, Hong JW, Hussein MA, Kim YO. Demographic characteristics of craniosynostosis patients in Asia. *Journal of Cranio-Maxillofacial Surgery.* 2018;46(4):674-678.
8. Cabrejo R, Lacadie C, Brooks E, et al. Understanding the learning disabilities linked to sagittal craniosynostosis. *Journal of Craniofacial Surgery.* 2019;30(2):497-502.
9. Care H, Horton J, Kearney A, et al. Introduction to the craniofacial collaboration UK: A developmental screening protocol at the United Kingdom's four highly specialized craniofacial centers. *Journal of Craniofacial Surgery.* 2019;30(1):83-86.
10. Chandler L, Allam O, Park KE, et al. Spring-Assisted Strip Craniectomy Versus Cranial Vault Remodeling: Long-Term Psychological, Behavioral, and Executive Function Outcomes. *Journal of Craniofacial Surgery.* 2020;31(7):2101-2105.
11. Chieffo D, Tamburrini G, Massimi L, et al. Long-term neuropsychological development in single-suture craniosynostosis treated early: Clinical article. *Journal of Neurosurgery: Pediatrics.* 2010;5(3):232-237.
12. Chieffo D, Arcangeli V, Bianchi F, et al. Single-suture craniosynostosis: is there a correlation between preoperative ophthalmological, neuroradiological, and neurocognitive findings? *Child's Nervous System.* 2020.
13. Chuang C, Rolison M, Yang JF, et al. Normalization of Speech Processing after Whole-Vault Cranioplasty in Sagittal Synostosis. *Journal of Craniofacial Surgery.* 2018;29(5):1132-1136.
14. Chuang C, Chaunzwa TL, Wu R, et al. Long-term neurocognitive outcomes in sagittal synostosis: The impact of reoperation. *Journal of Craniofacial Surgery.* 2021;32(1):58-61.
15. Da Costa AC, Walters I, Savarirayan R, Anderson VA, Wrennall JA, Meara JG. Intellectual outcomes in children and adolescents with syndromic and nonsyndromic craniosynostosis. *Plastic and Reconstructive Surgery.* 2006;118(1):175-181.
16. Da Costa AC, Anderson VA, Savarirayan R, et al. Neurodevelopmental functioning of infants with untreated single-suture craniosynostosis during early infancy. *Child's Nervous System.* 2012;28(6):869-877.
17. Da Costa AC, Anderson VA, Holmes AD, et al. Longitudinal study of the neurodevelopmental characteristics of treated and untreated nonsyndromic craniosynostosis in infancy. *Child's Nervous System.* 2013;29(6):985-995.
18. Engel M, Hoffmann J, Mühling J, Castrillón-Oberndorfer G, Seeberger R, Freudlsperger C. Subtotal cranial vault remodelling in anterior sagittal suture closure: Impact of age on surgical outcome. *International Journal of Oral and Maxillofacial Surgery.* 2012;41(10):1232-1237.

© 2021 Osborn AJ et al. *JAMA Network Open.*

19. Gewalli F, Da Silva Guimarães-Ferreira JP, Sahlin P, et al. Mental development after modified π procedure: Dynamic cranioplasty for sagittal synostosis. *Annals of Plastic Surgery.* 2001;46(4):415-420.
20. Hashim PW, Patel A, Yang JF, et al. The effects of whole-vault cranioplasty versus strip craniectomy on long-term neuropsychological outcomes in sagittal craniosynostosis. *Plastic and reconstructive surgery.* 2014;134(3):491-501.
21. Patel A, Yang JF, Hashim PW, et al. The impact of age at surgery on long-term neuropsychological outcomes in sagittal craniosynostosis. *Plast Reconstr Surg.* 2014;134(4):608e-617e.
22. Imahiyerobo TA, Johns AL, Christian EA, et al. Risk factors for preoperative developmental delay in patients with nonsyndromic sagittal craniosynostosis. *Plastic and Reconstructive Surgery.* 2019;143(1):133E-139E.
23. Kapp-Simon KA, Leroux B, Cunningham M, Speltz ML. Multisite study of infants with single-suture craniosynostosis: Preliminary report of presurgery development. *Cleft Palate-Craniofacial Journal.* 2005;42(4):377-384.
24. Ruiz-Correa S, Starr JR, Lin HJ, Kapp-Simon KA, Cunningham ML, Speltz ML. Severity of skull malformation is unrelated to presurgery neurobehavioral status of infants with sagittal synostosis. *Cleft Palate-Craniofacial Journal.* 2007;44(5):548-554.
25. Speltz ML, Kapp-Simon K, Collett B, et al. Neurodevelopment of infants with single-suture craniosynostosis: Presurgery comparisons with case-matched controls. *Plastic and Reconstructive Surgery.* 2007;119(6):1874-1881.
26. Starr JR, Kapp-Simon KA, Cloonan YK, et al. Presurgical and postsurgical assessment of the neurodevelopment of infants with single-suture craniosynostosis: Comparison with controls. *Journal of Neurosurgery.* 2007;107(2 SUPPL.):103-110.
27. Toth K, Collett B, Kapp-Simon KA, et al. Memory and response inhibition in young children with single-suture craniosynostosis. *Child Neuropsychology.* 2008;14(4):339-352.
28. Naumann HL, Haberkern CM, Pietila KE, et al. Duration of exposure to cranial vault surgery: Associations with neurodevelopment among children with single-suture craniosynostosis. *Paediatric Anaesthesia.* 2012;22(11):1053-1061.
29. Starr JR, Collett BR, Gaither R, et al. Multicenter study of neurodevelopment in 3-year-old children with and without single-suture craniosynostosis. *Archives of Pediatrics and Adolescent Medicine.* 2012;166(6):536-542.
30. Cradock MM, Gray KE, Kapp-Simon KA, Collett BR, Buono LA, Speltz ML. Sex differences in the neurodevelopment of school-age children with and without single-suture craniosynostosis. *Child's Nervous System.* 2015;31(7):1103-1111.
31. Speltz ML, Birgfeld C, Starr JR, Collett B, Kapp-Simon K. The effects of whole-vault cranioplasty versus strip craniectomy on long-term neuropsychological outcomes in sagittal craniosynostosis. *Plastic and Reconstructive Surgery.* 2015;135(3):646e-647e.
32. Speltz ML, Collett BR, Wallace ER, Kapp-Simon K. Behavioral adjustment of school-age children with and without single-suture craniosynostosis. *Plastic and Reconstructive Surgery.* 2016;138(2):435-445.
33. Kapp-Simon KA, Wallace E, Collett BR, Cradock MM, Crerand CE, Speltz ML. Language, learning, and memory in children with and without single-suture craniosynostosis. *Journal of Neurosurgery: Pediatrics.* 2016;17(5):578-588.
34. Collett BR, Kapp-Simon KA, Wallace E, Cradock MM, Buono L, Speltz ML. Attention and executive function in children with and without single-suture craniosynostosis. *Child Neuropsychology.* 2017;23(1):83-98.
35. Wallace ER, Collett BR, Kapp-Simon K, Starr JR, Birgfeld C, Speltz ML. Visuomotor Function in School-Age Children with Single-Suture Craniosynostosis. *Journal of Developmental and Behavioral Pediatrics.* 2016;37(6):483-490.

© 2021 Osborn AJ et al. *JAMA Network Open.*

36. Kapp-Simon KA, Figueroa A, Jocher CA, Schafer M. Longitudinal assessment of mental development in infants with nonsyndromic craniosynostosis with and without cranial release and reconstruction. *Plastic and Reconstructive Surgery.* 1993;92(5):831-839.
37. Kapp-Simon KA. Mental development and learning disorders in children with single suture craniosynostosis. *Cleft Palate-Craniofacial Journal.* 1998;35(3):197-203.
38. Kljajić M, Maltese G, Tarnow P, Sand P, Kölby L. The Cognitive Profile of Children with Nonsyndromic Craniosynostosis. *Plastic and Reconstructive Surgery.* 2019;143(5):1037e-1052e.
39. Kljajić M, Maltese G, Tarnow P, Sand P, Kölby L. Sustained attention and vigilance of children treated for sagittal and metopic craniosynostosis. *Child Neuropsychology.* 2020;26(4):475-488.
40. Korpilahti P, Saarinen P, Hukki J. Deficient language acquisition in children with single suture craniosynostosis and deformational posterior plagiocephaly. *Child's Nervous System.* 2012;28(3):419-425.
41. Lee MC, Shim KW, Park EK, Yun IS, Kim DS, Kim YO. Expansion and compression distraction osteogenesis based on volumetric and neurodevelopmental analysis in sagittal craniosynostosis. *Child's Nervous System.* 2015;31(11):2081-2089.
42. Lee MC, Shim KW, Yun IS, Park EK, Kim YO. Correction of Sagittal Craniosynostosis Using Distraction Osteogenesis Based on Strategic Categorization. *Plastic and Reconstructive Surgery.* 2017;139(1):157-169.
43. Magge SN, Westerveld M, Pruzinsky T, Persing JA. Long-term neuropsychological effects of sagittal craniosynostosis on child development. *Journal of Craniofacial Surgery.* 2002;13(1):99-104.
44. Mazzaferro DM, Naran S, Wes AM, Magee L, Taylor JA, Bartlett SP. Quality of life in adults with nonsyndromic craniosynostosis. *Plastic and Reconstructive Surgery.* 2018;141(6):1474-1482.
45. Moreno-Villagómez J, Yáñez-Téllez G, Prieto-Corona B, Seubert-Ravelo AN, García A. Cognitive performance of preschool children with different types of non-syndromic craniosynostosis. *Brain Impairment.* 2020:1-10.
46. Noetzel MJ, Marsh JL, Palkes H, Gado M. Hydrocephalus and mental retardation in craniosynostosis. *The Journal of Pediatrics.* 1985;107(6):885-892.
47. Salokorpi N, Savolainen T, Sinikumpu JJ, et al. Outcomes of 40 Nonsyndromic Sagittal Craniosynostosis Patients as Adults: A Case-Control Study with 26 Years of Postoperative Follow-up. *Operative Neurosurgery.* 2019;16(1):1-8.
48. Scheuerle J, Guilford AM, Habal MB. A Report of Behavioral Data on Three Groups of Patients With Craniofacial Disorders. *Journal of Craniofacial Surgery.* 2004;15(2):200-208.
49. Shipster C, Hearst D, Somerville A, Stackhouse J, Hayward R, Wade A. Speech, language, and cognitive development in children with isolated sagittal synostosis. *Developmental Medicine and Child Neurology.* 2003;45(1):34-43.
50. Speltz ML, Morton K, Goodell EW, Clarren SK. Psychological functioning of children with craniofacial anomalies and their mothers: Follow-up from late infancy to school entry. *Cleft Palate-Craniofacial Journal.* 1993;30(5):482-489.
51. Speltz ML, Endriga MC, Mouradian WE. Presurgical and postsurgical mental and psychomotor development of infants with sagittal synostosis. *Cleft Palate-Craniofacial Journal.* 1997;34(5):374-379.
52. Virtanen R, Korhonen T, Fagerholm J, Viljanto J. Neurocognitive sequelae of scaphocephaly. *Pediatrics.* 1999;103(4 I):791-795.
53. Care H, Kennedy-Williams P, Cunliffe A, et al. Preliminary Analysis From the Craniofacial Collaboration United Kingdom Developmental Outcomes in Children With Sagittal Synostosis. *Journal of Craniofacial Surgery.* 2019;30(6):1740-1744.

© 2021 Osborn AJ et al. *JAMA Network Open.*

54. Salokorpi N, Savolainen T, Sinikumpu JJ, Pirttiniemi P, Serlo W. Non-syndromic craniosynostosis patients operated between 1978 and 2000. How do they do as adults? *Child's Nervous System.* 2018;34(10):2031.

© 2021 Osborn AJ et al. *JAMA Network Open.*
